# Supplementary material for: Rad51 determines pathway usage in post-replication repair
Source: Nat Commun. 2026 Jan 10;17:1359. doi: 10.1038/s41467-025-68109-1 (PMC12876063; doi:10.1038/s41467-025-68109-1)
Supplement: Supplementary file 1 — Supplementary Information [file 41467_2025_68109_MOESM1_ESM.pdf]

Supplementary Information

Rad51 determines pathway usage in post-replication repair

Damon Meyer[1*#], Steven K. Gore[1#], Jie Liu[1#], Shannon J. Ceballos[1*#], Shih-Hsun Hung[1], Giordano Reginato[2], Maria I. Cano-Linares[3], Katarzyna H. Maslowska[4*], Florencia Villafañez[4*], Clare Fasching[1], Christopher Ede[1], Vincent Pagès[4], Felix Prado[3], Petr Cejka[2], and Wolf-Dietrich Heyer[1,5]

[1] Department of Microbiology & Molecular Genetics and [5] Department of Molecular & Cellular Biology, University of California, Davis, Davis CA 95616-8665, USA; [2] Institute for Research in Biomedicine, Università della Svizzera italiana (USI), Faculty of Biomedical Sciences, 6500 Bellinzona, Switzerland; [3] Centro Andaluz de Biología Molecular y Medicina Regenerativa – CABIMER; Consejo Superior de Investigaciones Científicas; Universidad de Sevilla; Universidad Pablo de Olavide; Seville, Spain; [4] Cancer Research Center of Marseille: Team DNA Damage and Genome Instability, CNRS, Aix Marseille Univ, Inserm, Institut Paoli-Calmettes, Marseille F-13009, France.

Address correspondence to:

Wolf-Dietrich Heyer, Department of Microbiology & Molecular Genetics, University of California, Davis, One Shields Ave., Davis, California 95616-8665, Tel. 530-752-3001; Fax. 530-752-3011; E-Mail: wdheyer@ucdavis.edu

Supplementary Tables 1-4

Supplementary References

Supplementary Figures 1-13

Supplementary Table 1: ***Saccharomyces cerevisiae*** **strains**

Number	Genotype	Source
WDHY1275	*MATa ade2-1 can1-100 his3-11,15 leu2-3, 112 trp1-1 ura3-1 rad54::KANMX*	This study
WDHY1611	*MATa his3-Δ1 leu2-3,112 trp1 ura3-52 pep4-3 rad51-Δ::KanMX* BJ strain for protein expression.	(1,2)
WDHY1636	*MATa ade2-1 can1-100 his3-11,15 leu2-3,112 trp1-1 ura3-1*	(3)
WDHY2217	*MATa ade2-1 can1-100 his3-11,15 leu2-3,112 trp1-1 ura3::loxP*	This study
WDHY2542	*MATa ade2-1 can1-100 his3-11,15 leu2-3, 112 trp1-1 ura3-1 rad51::KANMX*	This study
WDHY2543	*MATa ade2-1 can1-100 his3-11,15 leu2-3,112 trp1-1 ura3-1 rad51::KANMX*	This study
WDHY2544	*MATa ade2-1 can1-100 his3-11,15 leu2-3,112 trp1-1 ura3-1 rad51Δ::KANMX rad54Δ::KANMX*	This study
WDHY2546	*MATa ade2-1 can1-100 his3-11,15 leu2-3,112 trp1-1 or trp1-289 ura3-1 or ura 3-52 rad51Δ::KANMX rad54-3^{ts}(-C692Y)*	This study
WDHY2699	*MATa ade2-1 can1-100 HIS3 leu2-3,112 trp1-1 ura3-1 rad5-G535R*	This study
WDHY3162	*MATa ade2-1 CAN1 his3::(delta)5'his3(delta)3':: URA3 leu2-3, 112 trp1-1 ura3-1 rad51::LEU2 rad54::KANMX*	This study
WDHY3169	*MATa ade2-1 CAN1 his3::(delta)5'his3(delta)3':: URA3 leu2-3, 112 trp1-1 ura3-1 rad54::KANMX*	This study
WDHY3348	*MATa/MATα ade2-1/ade2-1 can1-100/can1-100 his3-11,15/his3-11,15 leu2(delta)EcoRI-URA3-HOcs(117)-leu2(delta)BstEII/leu2-3,112 trp1-1/trp1::KANMX-GALHO ura3-1/ura3-1 rad52::TRP1/RAD52*	This study
WDHY3349	*MATa/MATα ade2-1/ade2-1 can1-100/can1-100 HIS3/his3-11,15 leu2(delta)EcoRI-URA3-HOcs(117)-leu2(delta)BstEII/leu2-3,112 trp1-1/trp1::KANMX-GALHO ura3-1/ura3-1 rad54::KANMX/RAD54*	This study
WDHY3383	*MATa/MATα ade2-1/ade2-1 can1-100/can1-100 his3Δ200/his3-11,15 leu2(delta)EcoRI-URA3-HOcs(117)-leu2(delta)BstEII/leu2-3,112 trp1-1/trp1::KANMX-GALHO ura3-1/ura3-1*	This study
WDHY3385	*MATa/MATα ade2-1/ade2-1 can1-100/can1-100 his3-11,15/his3-11,15 leu2(delta)EcoRI-URA3-HOcs(117)-leu2(delta)BstEII/leu2-3,112 trp1-1/trp1::KANMX-GALHO ura3-1/ura3::TRP1 RAD51/rad51-K305N*	This study
WDHY3386	*MATa/MATα ade2-1/ade2-1 can1-100/can1-100 HIS3/his3-11,15 leu2(delta)EcoRI-URA3-HOcs(117)-leu2(delta)BstEII/leu2-3,112 trp1-1/trp1::KANMX-GALHO ura3-1/ura3-1 rad51E135D/RAD51*	This study
WDHY3462	*MATa/MATα ade2-1/ade2-1 can1-100/can1-100 his3-11,15/his3-11,15 leu2(delta)EcoRI-URA3-HOcs(117)-leu2(delta)BstEII/leu2-3,112 trp1-1/trp1::KANMX-GALHO ura3-1/ura3::TRP1 RAD51/rad51-E135D RAD54/rad54::KANMX*	This study
WDHY3463	*MATa/MATα ade2-1/ade2-1 can1-100/can1-100 his3-11,15/his3-11,15 leu2(delta)EcoRI-URA3-HOcs(117)-leu2(delta)BstEII/leu2-3,112 trp1-1/trp1::KANMX-GALHO ura3-1/ura3::TRP1 RAD51/rad51-K305N RAD54/rad54::KANMX*	This study
WDHY3536	*MATa ade2-1 can1-100 HIS3 trp1-1 leu2-3,112 ura3-1 rad5-G535R rad51-E135D*	This study
WDHY3539	*MATa ade2-1 can1-100 his3-11,15 TRP1 leu2-3,112 ura3-1 rad5-G535R rad51-E135D rad54::KANMX*	This study
WDHY3546	*MATα ade2-1 can1-100 his3-11,15 trp1-1 leu2-3,112 ura3-1 rad51-E135D rad54::KANMX*	This study
WDHY3547	*MATα ade2-1 can1-100 his3-11,15 trp1-1 leu2-3,112 ura3-1 rad51-E135D rad54::KANMX*	This study
WDHY3548	*MATa ade2-1 can1-100 his3-11,15 trp1-1 leu2-3,112 ura3-1 rad51-E135D*	This study

WDHY3555	*MATa ade2-1 can1-100 leu2-3,112 ura3-1 pol30-K127R,K164R*	This study
WDHY3557	*MATa ade2-1 can1-100 his3-11,15 leu2-3,112 ura3-1 rad51-K305N pol30-K127R,K164R*	This study
WDHY3561	*MATα ade2-1 can1-100 his3-11,15 leu2-3,112 ura3-1 rad51-E135D pol30-K127R,K164R*	This study
WDHY3563	*MATα ade2-1 can1-100 leu2-3,112 ura3-1 rad54::KANMX pol30-K127R,K164R*	This study
WDHY3568	*MATa ade2-1 can1-100 his3-11,15 leu2-3,112 ura3-1 rad51-K305N rad54::KANMX pol30-K127R,K164R*	This study
WDHY3570	*MATa ade2-1 can1-100 his3-11,15 leu2-3,112 ura3-1 rad51-E135D rad54::KANMX pol30-K127R,K164R*	This study
WDHY3572	*MATa ade2-1 can1-100 his3-11,15 trp1-1 leu2-3,112 ura3-1 rad51-K305N rad54::KANMX*	This study
WDHY3578	*MATa ade2-1 can1-100 his3-11,15 trp1-1 leu2-3,112 ura3-1 rad5-G535R rad51-K305N*	This study
WDHY3581	*MATα ade2-1 can1-100 his3-11,15 TRP1 leu2-3,112 ura3-1 rad5-G535R rad51-K305N rad54::KANMX*	This study
WDHY3584	*MATa ade2-1 can1-100 trp1-1 leu2-3,112 ura3-1 rev3::KANMX*	This study
WDHY3588	*MATα ade2-1 can1-100 trp1-1 leu2-3,112 ura3-1 his3-11,15 rad54::KANMX rev3::KANMX*	This study
WDHY3589	*MATa ade2-1 can1-100 trp1-1 leu2-3,112 ura3-1 rad51-K305N rev3::KANMX*	This study
WDHY3780	*MATα ade2-1 can1-100 trp1-1 leu2-3,112 ura3-1 his3-11,15 rad51::KANMX rad54::KANMX pol30-K127R,K164R*	This study
WDHY3783	*MATα ade2-1 can1-100 trp1::KANMX-GAL-HO leu2-3,112 ura3-1 his3-11,15 rad51::LEU2 pol30-K127R,K164R*	This study
WDHY3784	*MATa ade2-1 can1-100 leu2-3,112 ura3-1 his3-11,15 rad51-E135D rev3::KANMX*	This study
WDHY3785	*MATα ade2-1 can1-100 trp1-1 leu2-3,112 his3-11,15 trp1-1 rad51-E135D rad54:KANMX rev3::KANMX*	This study
WDHY3787	*MATa ade2-1 can1-100 trp1-1 leu2-3,112 his3-11,15 trp1-1 rad51::LEU2 rev3::KANMX*	This study
WDHY3788	*MATα ade2-1 can1-100 trp1-1 leu2-3,112 ura3-1 his3-11,15 rad51::LEU2 rad54::KANMX rev3::KANMX*	This study
WDHY3789	*MATa ade2-1 can1-100 trp1-1 leu2-3,112 ura3-1 his3-11,15 rad51-K305N rad54::KANMX rev3::KANMX*	This study
WDHY3855	*MATα ade2-1 can1-100 HIS3 leu2-3,112 trip1-1 ura3-1 mms2::KANMX*	This study
WDHY3898	*MATa ade2-1 can1-100 his3-11,15 leu2-3,112 TRP1 ura3-1 rad51::HIS3*	This study
WDHY3915	*MATa/MATα ade2-1/ade2-1 can1-100/can1-100 his3-11,15/his3-11,15 leu2(delta)EcoRI-URA3-HOcs(117)-leu2(delta) BstEII/leu2-3,112 TRP1/trp1::KANMX-GALHO ura3-1/ura3-1 rad51::HIS3/RAD51*	This study
WDHY3952	*MATα ade2-1 can1-100 HIS3 leu2-3,112 trip1-1 ura3-1 mms2::KANMX rad51::HIS3*	This study
WDHY3956	*MATα ade2-1 can1-100 HIS3 leu2-3,112 trip1-1 ura3-1 mms2::KANMX rad54::KANMX*	This study
WDHY3958	*MATα ade2-1 can1-100 HIS3 leu2-3,112 trip1-1 ura3-1 mms2::KANMX rad51-K305N*	This study
WDHY3959	*MATα ade2-1 can1-100 his3-11, 15 leu2-3,112 ura3-1 trp1-1 rad51::HIS3 rad54::KanMX*	This study
WDHY3960	*MATa ade2-1 can1-100 his3-11, 15 leu2-3,112 ura3-1 trp1-1*	This study
WDHY3961	*MATα ade2-1 can1-100 his3-11 15 leu2-3,112 ura3-1 trp1-1 rad54::kanMX*	This study
WDHY3962	*MATα ade2-1 can1-100 his3-11,15 trp1-1 leu2-3,112 ura3-1*	

	rad51-K305N	This study
WDHY3963	*MATa ade2-1 can1-100 his3-11,15 trp1-1 leu2-3,112 ura3-1 rad51-K305N rad54::KanMX*	This study
WDHY4204	*MATa/MATα ade2-1/ade2-1 CAN1/can1-100 HIS3/his3-11,15 leu2-3,112/leu2-3,112 trp1-1/trp1-1 ura3-1/ura3-1 hxt13::URA3/HXT13 rad51::HIS3/RAD51*	This study
WDHY4205	*MATa/MATα ade2-1/ade2-1 CAN1/can1-100 HIS3/his3-11,15 leu2-3,112/leu2-3,112 trp1-1/trp1-1 ura3-1/ura3-1 hxt13::URA3/HXT13 rad54::KANMX/RAD54*	This study
WDHY4207	*MATa/MATα ade2-1/ade2-1 CAN1/can1-100 HIS3/his3-11,15 leu2-3,112/leu2-3,112 trp1-1/trp1-1 ura3-1/ura3-1 hxt13::URA3/HXT13 rad51-E135D/RAD51*	This study
WDHY4208	*MATa/MATα ade2-1/ade2-1 CAN1/can1-100 HIS3/his3-11,15 leu2-3,112/leu2-3,112 trp1-1/trp1-1 ura3-1/ura3-1, hxt13::URA3/HXT13 rad51-K305N/RAD51*	This study
WDHY4209	*MATa/MATα ade2-1/ade2-1 CAN1/can1-100 HIS3/his3-11,15 leu2-3,112/leu2-3,112 trp1-1/trp1-1 ura3-1/ura3-1 hxt13::URA3/HXT13 rad51-E135D/RAD51 rad54::KANMX/RAD54*	This study
WDHY4210	*MATa/MATα ade2-1/ade2-1 CAN1/can1-100 HIS3/his3-11,15 leu2-3,112/leu2-3,112 trp1-1/trp1-1, ura3-1/ura3-1 hxt13::URA3/HXT13 rad51-K305N/RAD51 rad54::KANMX/RAD54*	This study
WDHY4564	*MATa/MATα ade2-1/ade2-1 CAN1/can1-100 HIS3/his3-11,15 leu2-3,112/leu2-3,112 TRP1/trp1-1 ura3-1/ura3-1 mms2::KANMX/MMS2 rad51-E135D/RAD51 rad54::KANMX/RAD54*	This study
WHY4635	*MATα ade2-1 can1-100 his3-11,15 leu2-3,112 trp1-1 ura3::loxP rad51::KANMX*	This study
WDHY5169	*MATa ade2-1 can1-100 his3-11,15 leu2-3,112 trp1-1 ura3-1 rad51-E135D mms2::KANMX*	This study
WDHY5509	*MATa-inc ura3::LY-HOcs, lys2::LYover0, trp1::GAL-HO-hphMX, his3D200, can1-100, leu2-3,112, ade2-1, RAD5*	This study
WDHY6127	*MATa ade2-1 can1-100 his3-11,15 leu2-3,112 trp1-1 ura3-1 rad51-E135D rad54::KANMX mms2::KANMX*	This study
WDHY6283	*MATa ade2-1 can1-100 ura3-1 his3-11 leu2-3,112 RAD5 trp1::GAL-HO-hphMX, rad51-E135D*	This study
WDHY6288	*MATa-inc ura3::LY-HOcs, lys2::LYover0, trp1::GAL-HO-hphMX, his3D200, can1-100, leu2-3,112, ade2-1, RAD5 rad51-K305N*	This study
WDHY6298	*MATa-inc lys2::NatMX4 AVT2::lys-HOcs:: KanMX6 ade3::GAL-HO C0S9::TRP1-ys2 Ch XI 15 kb donor RAD5*	(4)
WDHY6299	*MATa-inc lys2::NatMX4 AVT2::lys-HOcs:: KanMX6 ade3::GAL-HO C0S9::TRP1-ys2 Ch XI 15 kb donor RAD5 rad51-K305N*	This study
WDHY6304	*MATa-inc lys2::NatMX4 AVT2::lys-HOcs:: KanMX6 ade3::GAL-HO C0S9::TRP1-ys2 Ch XI 15 kb donor RAD5 rad51-E135D*	This study
wR51MN-2	*MATa ade2-1 can1-100 his3-11,15 leu2-3,112 trp1-1 ura3-1 RAD51-MN::HIS3*	(5)
wR51-135MN	*MATa ade2-1 can1-100 leu2-3,112 trp1-1 ura3::loxP his3-11,15 rad51-E135D-MN::HIS3*	This study
wR51-305MN	*MATa ade2-1 can1-100 leu2-3,112 trp1-1 ura3::loxP his3-11,15 rad51-K305N-MN::HIS3*	This study
W303sgs1	*MATa ade2-1 can1-100 his3-11,15 leu2-3,112 trp1-1 ura3-1 sgs1::KANMX*	(6)
W303sr305	*MATa ade2-1 can1-100 his3-11,15 leu2-3,112 trp1-1 ura3 rad51-K305N sgs1::KANMX*	This study
W303sr135	*MATa ade2-1 can1-100 his3-11,15 leu2-3,112 trp1-1 ura3 rad51-E135D sgs1::KANMX*	This study

Unless otherwise noted, all strains have the W303 background and are wild type for *RAD5*.

SC53	*MATa his3-Δ1 leu2-3,112 trp1-Δ ura3-Δ met25-Δ rad14-Δ phr1-Δ msh2Δ::hisG VI(167260–167265):: (lox66-3'lacZ-MET25/lag)*	(7)
SC55	*MATa his3-Δ1 leu2-3,112 trp1-Δ ura3-Δ met25-Δ rad14-Δ phr1-Δ msh2Δ::hisG VI(167260–167265):: (lox66-3'lacZ-MET25/lead)*	(7)
SC254	*MATa his3-Δ1 leu2-3,112 trp1-Δ ura3-Δ met25-Δ rad14-Δ phr1-Δ msh2Δ::hisG rad51Δ::KAN VI(167260–167265):: (lox66-3'lacZ-MET25/lag)*	(7)
SC255	*MATa his3-Δ1 leu2-3,112 trp1-Δ ura3-Δ met25-Δ rad14-Δ phr1-Δ msh2Δ::hisG rad51Δ::KAN VI(167260–167265):: (lox66-3'lacZ-MET25/lead)*	(7)
SC844	*MATa his3-Δ1 leu2-3,112 trp1-Δ ura3-Δ met25-Δ rad14-Δ phr1-Δ msh2Δ::hisG rad51-E135D VI(167260–167265):: (lox66-3'lacZ-MET25/lag)*	This study
SC845	*MATa his3-Δ1 leu2-3,112 trp1-Δ ura3-Δ met25-Δ rad14-Δ phr1-Δ msh2Δ::hisG rad51E135D VI(167260–167265):: (lox66-3'lacZ-MET25/lead)*	This study
SC868	*MATa his3-Δ1 leu2-3,112 trp1-Δ ura3-Δ met25-Δ rad14-Δ phr1-Δ msh2Δ::hisG rad51-K305N VI(167260–167265):: (lox66-3'lacZ-MET25/lag)*	This study
SC869	*MATa his3-Δ1 leu2-3,112 trp1-Δ ura3-Δ met25-Δ rad14-Δ phr1-Δ msh2Δ::hisG rad51-K305N VI(167260–167265):: (lox66-3'lacZ-MET25/lead)*	This study

All SC strains share the EMY74.7 background (8).

WDHY668	*MATa/α ura3-52/ ura3-52 trp1/ trp1 leu2∆1/ leu2∆1 his3∆200/ his3∆200 pep4::HIS3/ pep4::HIS3 prb1∆1.6R/ prb1∆1.6R can1/ can1 GAL/ GAL*	*(9)*

Strain for protein purification with BJ background (2).

Supplementary Table 2: Plasmids

Number	Description	Source
WDH647	pR51.3 with wild type *RAD51* for protein expression	(10)
pWDH951	pR51.3 with *rad51-E135D* for protein expression	This study
pWDH952	pR51.3 with *rad51-K305N* for protein expression	This study
pWDH953	YEp351-*rad51-K305N* +1,000 bp up/downstream of ORF	This study
pWDH954	YEp351-*rad51-E135D* +1,000 bp up/downstream of ORF	This study
pWDH957	YEp351-*RAD51* +1,000 bp up/downstream of ORF	This study
pWDH958	YEp351 *LEU2* amp^R	(11)
pFB-MBP-Sgs1-his	Expression of MBP- and His-tagged Sgs1 in insect cells	(12)
pFB-Exo1-FLAG	Expression of FLAG-tagged Exo1 in insect cells	(13)
pGAL:FLAG-DNA2-his	Expression of FLAG-, HA- and His-tagged Dna2 in yeast	(14)
p11d–tRPA	Expression of Rfa1, Rfa2 and Rfa3 in bacteria	(15)

Supplementary Table 3: Oligonucleotides

Number	Sequence and use	Source
olWDH566	5′- ATGGCAGCACTGCATAATTCTCTTACTGTCATGCCATCCGTAAGATG CTTTTCTGTGACTGGTGAGTACTCAACCAAGTCATTCTGAGAATAGTG<br>D-loop assay (Fig. S9D)	Operon
olWDH632	5′- AATGGACGGTAAATGTTGGA<br>Forward primer for random *RAD51* mutagenesis and *in vivo* recombination, ~400 nt upstream	Operon
olWDH633	5′- AACGTCGAAACGAAGACAAG<br>Reverse primer for random *RAD51* mutagenesis and *in vivo* recombination, ~ 400 nt downstream	Operon
olWDH830	5′- CCCGAGCTCCTCAGCGAAGTCGTGAAACTCGGA<br>Reverse primer 1,000 downstream *RAD51* open reading frame with *Sac*I site	Invitrogen
olWDH1351	5′- AATTCCAGCTGACCACCATGATGTCTCAAGTTCAAGAACAA<br>Forward *RAD51* adaptamer for mutant integration	Invitrogen
olWDH1352	5′- GATCCCCGGGAATTGCCATGAGAATTGAAAGTAAACCTGTG<br>Reverse *RAD51* adaptamer for mutant integration	Invitrogen
olWDH1355	5′- CCCAAGCTTCCGCAATAAAGGGCTTCCCGGACT<br>Forward primer 1,000 upstream of *RAD51* open reading frame with *Hind*III site	Invitrogen
olWDH1357	5′- TGCAATATCAACGGTACCCTTAGT<br>Internal *K. lactis URA3* primer	Invitrogen
olWDH1358	5′- GGTCCATACATTTGCCTTTTGAAA<br>Internal *K. lactis URA3* primer	Invitrogen
olWDH2086	5′- /Cy5/AACGACGTTTGGTCAGTTCCATCAACATCATAGCCAGATGCC CAGAGATTAGAGCGCATGACAAGTAAAGGACGGTTGTCAGCGTCATAAGAG GTTTTAC<br>100 nt Cy5 labeled oligonucleotide for EMSA (Fig. 4)	IDT
olWDH2182	5′- /Cy5/TTGATGGAACTGACCAAACGTCGTT<br>25 nt oligonucleotide complementary to 5’ end of olWDH2182 form the tailed substrate used in the D-loop assay (Fig. 5D, E)	IDT
olWDH2183	5′- AACGACGTTTGGTCAGTTCCATCAACATCATAGCCAGATGCCCAGAG ATTAGAGCGCATGACAAGTAAAGGACGGTTGTCAGCGTCATAAGAGGTTTTAC<br>100 nt oligonucleotide for D-loop assay (Figs. 5, S9A-C)	IDT
olWDH2184	5′- GTAAAACCTCTTATGACGCTGACAACCGTCCTTTACTTGTCATGCGCT CTAATCTCTGGGCATCTGGCTATGATGTTGATGGAACTGACCAAACGTCGTT<br>100 nt oligonucleotide complement for olWDH2086 EMSA (Fig. 4)	IDT
BIO100C	5′- GATGCAGGAGGCTGCTACGACCATGGCAGAAGATTATGAGGTGGAGT ACGCGCCCGGGGAGCCCAAGGGCACGCCCTGGCACCCGCACCGCGGCACTTAC<br>100 nt oligonucleotide for the 100 bp substrate used in helicase and nuclease assays (Figs. 6, S10)	Eurogentec
BIO100	5′- G**T**AAGTGCCGCGGTGCGGGTGCCAGGGCGTGCCCTTGGGCTCCCCGG GCGCGTACTCCACCTCATAATCTTCTGCCATGGTCGTAGCAGCCTCCTGCATC<br>100 nt oligonucleotide for the 100 bp substrate used in helicase and nuclease assays (the bold **T** represent the position of biotin-conjugated T) (Figs. 6, S10)	Eurogentec

Supplementary Table 4: Confidence intervals for the median. Two sided Symmetric – 95% or better

Nonparametric two-sided confidence intervals for the median of a continuous distribution based on order statistics. Shown are the narrowest symmetric intervals whose confidence coefficient is 95% or better.

Key

N Sample size

L U Order statistics defining the Lower and Upper endpoints

P (16) Probability the interval does not cover the true median (never exceeds 0.05)

N	L	U	P{miss}	N	L	U	P{miss}	N	L	U	P{miss}
1	.	.	.	41	14	28	0.02753	81	32	50	0.04483
2	.	.	.	42	15	28	0.04356	82	32	51	0.03524
3	.	.	.	43	15	29	0.03154	83	33	51	0.04752
4	.	.	.	44	16	29	0.04877	84	33	52	0.03753
5	.	.	.	45	16	30	0.03570	85	33	53	0.02946
6	1	6	0.03125	46	16	31	0.02590	86	34	53	0.03985
7	1	7	0.01563	47	17	31	0.03999	87	34	54	0.03142
8	1	8	0.00781	48	17	32	0.02930	88	35	54	0.04221
9	2	8	0.03906	49	18	32	0.04438	89	35	55	0.03342
10	2	9	0.02148	50	18	33	0.03284	90	36	55	0.04460
11	2	10	0.01172	51	19	33	0.04887	91	36	56	0.03545
12	3	10	0.03857	52	19	34	0.03648	92	37	56	0.04701
13	3	11	0.02246	53	19	35	0.02701	93	37	57	0.03751
14	3	12	0.01294	54	20	35	0.04022	94	38	57	0.04945
15	4	12	0.03516	55	20	36	0.03003	95	38	58	0.03961
16	4	13	0.02127	56	21	36	0.04405	96	38	59	0.03155
17	5	13	0.04904	57	21	37	0.03314	97	39	59	0.04173
18	5	14	0.03088	58	22	37	0.04794	98	39	60	0.03336
19	5	15	0.01921	59	22	38	0.03634	99	40	60	0.04388
20	6	15	0.04139	60	22	39	0.02734	100	40	61	0.03520
21	6	16	0.02660	61	23	39	0.03962	101	41	61	0.04604
22	6	17	0.01690	62	23	40	0.03002	102	41	62	0.03707
23	7	17	0.03469	63	24	40	0.04296	103	42	62	0.04823
24	7	18	0.02266	64	24	41	0.03277	104	42	63	0.03896
25	8	18	0.04329	65	25	41	0.04635	105	42	64	0.03130
26	8	19	0.02896	66	25	42	0.03558	106	43	64	0.04087
27	8	20	0.01916	67	26	42	0.04980	107	43	65	0.03295
28	9	20	0.03570	68	26	43	0.03846	108	44	65	0.04281
29	9	21	0.02412	69	26	44	0.02949	109	44	66	0.03462
30	10	21	0.04277	70	27	44	0.04139	110	45	66	0.04476
31	10	22	0.02945	71	27	45	0.03193	111	45	67	0.03631
32	10	23	0.02006	72	28	45	0.04437	112	46	67	0.04674
33	11	23	0.03508	73	28	46	0.03442	113	46	68	0.03802
34	11	24	0.02431	74	29	46	0.04739	114	47	68	0.04872
35	12	24	0.04096	75	29	47	0.03695	115	47	69	0.03975
36	12	25	0.02882	76	29	48	0.02863	116	47	70	0.03227
37	13	25	0.04703	77	30	48	0.03954	117	48	70	0.04150
38	13	26	0.03355	78	30	49	0.03079	118	48	71	0.03379
39	13	27	0.02370	79	31	49	0.04217	119	49	71	0.04327
40	14	27	0.03848	80	31	50	0.03299	120	49	72	0.03532

Table Entries Can (usually) **Be Calculated**

With only TWO exceptions, the table entries agree with this formula:

L = floor[(N+1)/2 - 0.9789 sqrt(N)]

These two exceptions are --

N = 17	L = 5	U = 13	the formula obtains L= 4 (U=14)
N = 67	L = 26	U = 42	the formula obtains L=25 (U=43)

In both cases the formula is conservative.

Beyond table, the formula works from N=6 through N=283 save for the two execeptions listed above.

For 284 and beyond this formula suffices for practical purposes:

L = floor[(N+1)/2 - 0.9800 sqrt(N)]

Example:

For a random sample of	53	67	85	98	30	37	69	77	79	45	49	106
First order the sample	30	37	45	49	53	67	69	77	79	85	98	106
(order number)	1	2	3	4	5	6	7	8	9	10	11	12

For N=12, the table shows L=3, U=10, P{miss}=0.03857

45 to 85 is a 96.142% confidence interval for the median.

This table is public domain.

Entries of this table were calculated with APL programs written by William Knight

University of New Brunswick, Canada.
knight@unb.ca

Supplementary references

1. Zhang, X.P., Galkin, V.E., Yu, X., Egelman, E.H. and Heyer, W.D. (2009) Loop 2 in *Saccharomyces cerevisiae* Rad51 protein regulates filament formation and ATPase activity. *Nucleic Acids Res.*, **37**, 158-171.
2. Jones, E.W. (1991) Tackling the protease problem in *Saccharomyces cerevisiae*. *Methods Enzymol.*, **194**, 428-453.
3. Thomas, B.J. and Rothstein, R. (1989) The genetic control of direct-repeat recombination in *Saccharomyces*: the effect of *rad52* and *rad1* on mitotic recombination at *GAL10*, a transcriptionally regulated gene. *Genetics*, **123**, 725-738.
4. Donnianni, R.A. and Symington, L.S. (2013) Break-induced replication occurs by conservative DNA synthesis. *Proc Natl Acad Sci USA*, **110**, 13475-13480.
5. Cabello-Lobato, M.J., Gonzalez-Garrido, C., Cano-Linares, M.I., Wong, R.P., Yanez-Vilchez, A., Morillo-Huesca, M., Roldan-Romero, J.M., Vicioso, M., Gonzalez-Prieto, R., Ulrich, H.D. *et al.* (2021) Physical interactions between MCM and Rad51 facilitate replication fork lesion bypass and ssDNA gap filling by non-recombinogenic functions. *Cell Rep*, **36**, 109440.
6. Gonzalez-Prieto, R., Munoz-Cabello, A.M., Cabello-Lobato, M.J. and Prado, F. (2013) Rad51 replication fork recruitment is required for DNA damage tolerance. *EMBO J.*, **32**, 1307-1321.
7. Maslowska, K.H., Laureti, L. and Pages, V. (2019) iDamage: a method to integrate modified DNA into the yeast genome. *Nucleic Acids Res.*, **47**,e124.
8. Johnson, R.E., Kovvali, G.K., Prakash, L. and Prakash, S. (1998) Role of yeast Rth1 nuclease and its homologs in mutation avoidance, DNA repair, and DNA replication. *Curr. Genet.*, **34**, 21-29.
9. Solinger, J.A., Lutz, G., Sugiyama, T., Kowalczykowski, S.C. and Heyer, W.-D. (2001) Rad54 protein stimulates heteroduplex DNA formation in the synaptic phase of DNA strand exchange *via* specific interactions with the presynaptic Rad51 nucleoprotein filament. *J. Mol. Biol.*, **307**, 1207-1221.
10. Sung, P. (1994) Catalysis of ATP-dependent homologous DNA pairing and strand exchange by yeast RAD51 protein. *Science*, **265**, 1241-1243.
11. Hill, J.E., Myers, A.M., Koerner, T.J. and Tzagoloff, A. (1986) Yeast/E. coli shuttle vectors with multiple unique restriction sites. *Yeast*, **2**, 163-167.
12. Cejka, P. and Kowalczykowski, S.C. (2010) The full-length *Saccharomyces cerevisiae* Sgs1 protein is a vigorous DNA helicase that preferentially unwinds Holliday junctions. *J Biol Chem*, **285**, 8290-8301.
13. Cannavo, E., Cejka, P. and Kowalczykowski, S.C. (2013) Relationship of DNA degradation by *Saccharomyces cerevisiae* Exonuclease 1 and its stimulation by RPA and Mre11-Rad50-Xrs2 to DNA end resection. *Proc Natl Acad Sci USA*, **110**, E1661-E1668.
14. Cejka, P., Cannavo, E., Polaczek, P., Masuda-Sasa, T., Pokharel, S., Campbell, J.L. and Kowalczykowski, S.C. (2010) DNA end resection by Dna2-Sgs1-RPA and its stimulation by Top3-Rmi1 and Mre11-Rad50-Xrs2. *Nature*, **467**, 112-116.
15. Binz, S.K., Dickson, A.M., Haring, S.J. and Wold, M.S. (2006) Functional assays for replication protein A (RPA). *Methods Enzymol*, **409**, 11-38.
16. Meddows, T.R., Savory, A.P., Grove, J.I., Moore, T. and Lloyd, R.G. (2005) RecN protein and transcription factor DksA combine to promote faithful recombinational repair of DNA double-strand breaks. **57**, 97-110.
17. Liu, J., Gore, S.K. and Heyer, W.D. (2025) Local structural dynamics of Rad51 protomers revealed by cryo-electron microscopy of Rad51-ssDNA filaments. *Nucleic Acids Res.*, **53**,gkaf052.

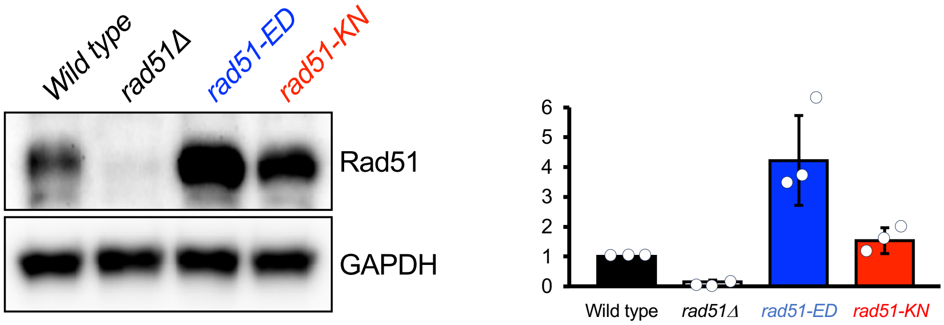

Supplementary Figure 1. Rad51-E135D and Rad51-KN show elevated steady state protein levels. Immunoblot analysis of Rad51 protein levels in extracts from wild type *RAD51* (WDHY1636), *rad51Δ* (WDHY2543), *rad51-ED* (WDHY3548), and *rad51-KN* (WDHY3962) strains. The amounts of Rad51 protein normalized to the glyceraldehyde-3-phosphate dehydrogenase (GAPDH) loading control relative to wild-type are shown as means from n=3. Source data are provided as a Source Data file.

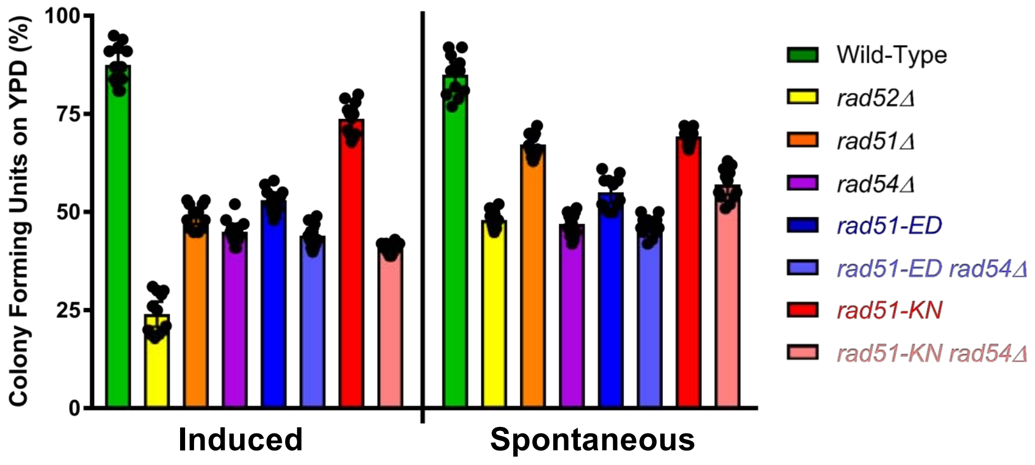

Supplementary Figure 2. Viability data for Figure 2 after DSB induction (induced) or without DSB induction (spontaneous). The average number of colonies that grew on YPD was plotted to ascertain efficiency of plating and viability before and after DSB induction. Cells were counted in a hemocytometer and plated on YPD. Strains were freshly dissected from diploid strains and single spore clones were assayed. Diploids allowed for multiple strains to be created by dissection. Wildtype (WDHY3383), *rad52Δ* (WDHY3348), *rad51Δ* (WDHY3915), *rad51-KN* (WDHY3385), *rad51-KN rad54Δ* (WDHY3463), *rad51-ED* (WDHY3386), *rad51-ED rad54Δ* (WDHY3462), *rad54Δ* (WDHY3349). Shown are means and error bars represent 1 standard deviation, n=12. Source data are provided as a Source Data file.

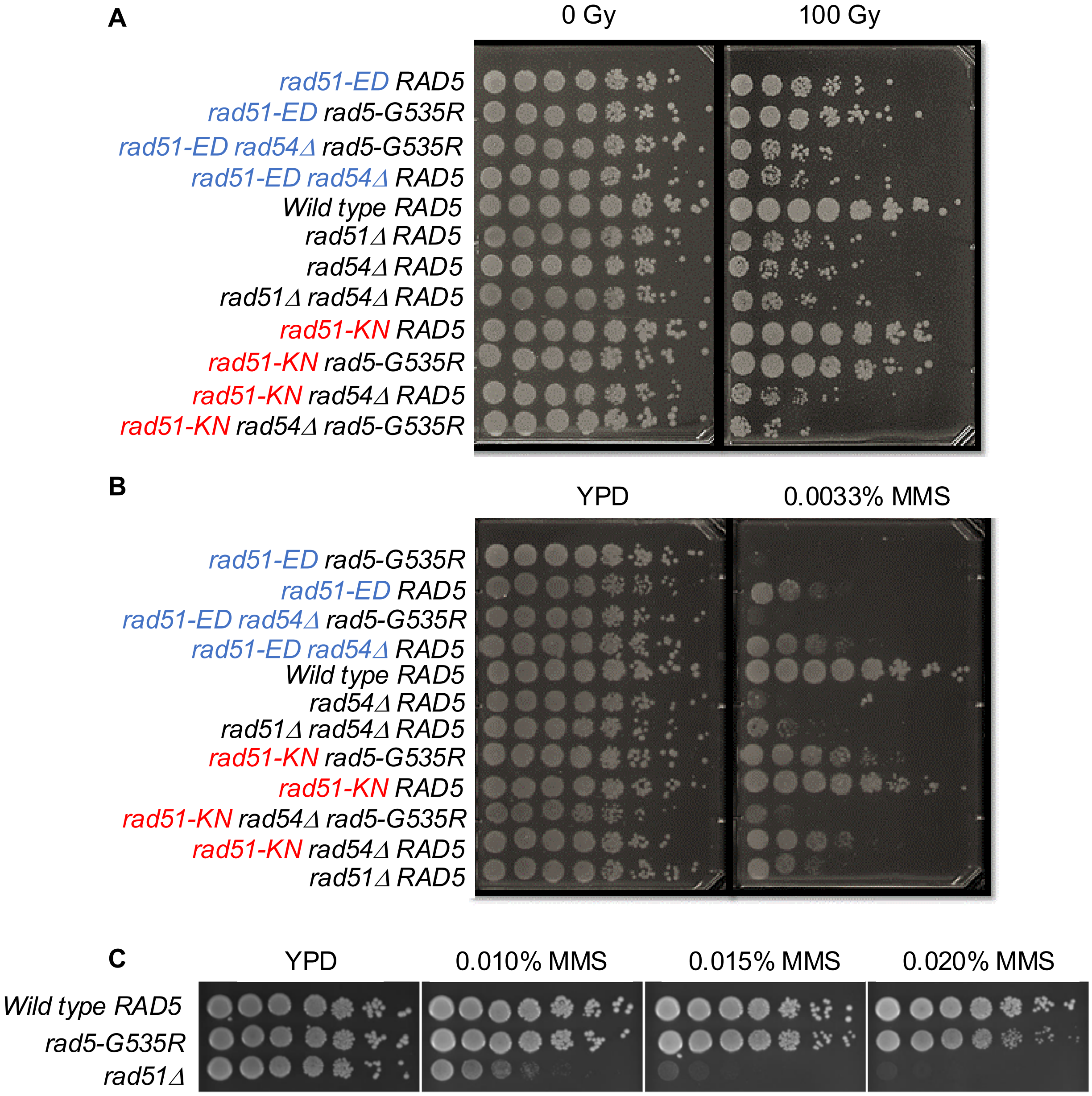

Supplementary Figure 3. IR and MMS sensitivities of *rad51* mutants. A, B. Serial dilutions of strains with chromosomally integrated mutations: Wild-type *RAD5* (WDHY1636), *rad54Δ RAD5* (WDH1275), *rad51Δ RAD5* (WDHY2542), *rad51Δ rad54Δ RAD5* (WDHY2544), *rad51-KN RAD5* (WDHY3962), *rad51-KN rad54Δ RAD5* (WDHY3572), *rad51-KN rad5-G535R* (WDHY3578), *rad51-KN rad54Δ rad5-G535R* (WDHY3581), *rad51-ED RAD5* (WDHY3548), *rad51-ED rad54Δ RAD5* (WDHY3546), *rad51-ED rad5-G535R* (WDHY3536), *rad51-ED rad54Δ rad5-G535R* (WDHY3539) to monitor sensitivity to 100 Gy ionizing radiation (**A**) or 0.0033% MMS (**B**). **C.** Serial dilutions of wild type (WDHY1636), *rad5-G535R* (WDHY2699), and *rad51Δ* (WDHY4635) strains were grown on plates containing the indicated MMS concentrations. Source data are provided as a Source Data file.

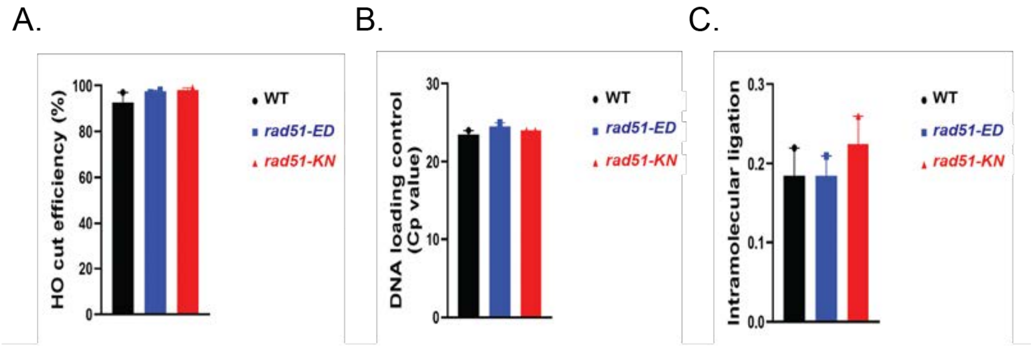

Supplementary Figure 4. Control experiments for the DLC assay in Figure 3. A. Plot of individual values of the HO cutting efficiency of the DLC assay of each strain. **B.** Plot of individual values of the DNA loading control of the DLC assay of each strain. **C**. Plot of individual values of the intramolecular ligation control of the DLC assay of each strain. Shown are mans of n=3 (wt) and n=2 (*rad51-ED, rad51-KN*). Source data are provided as a Source Data file.

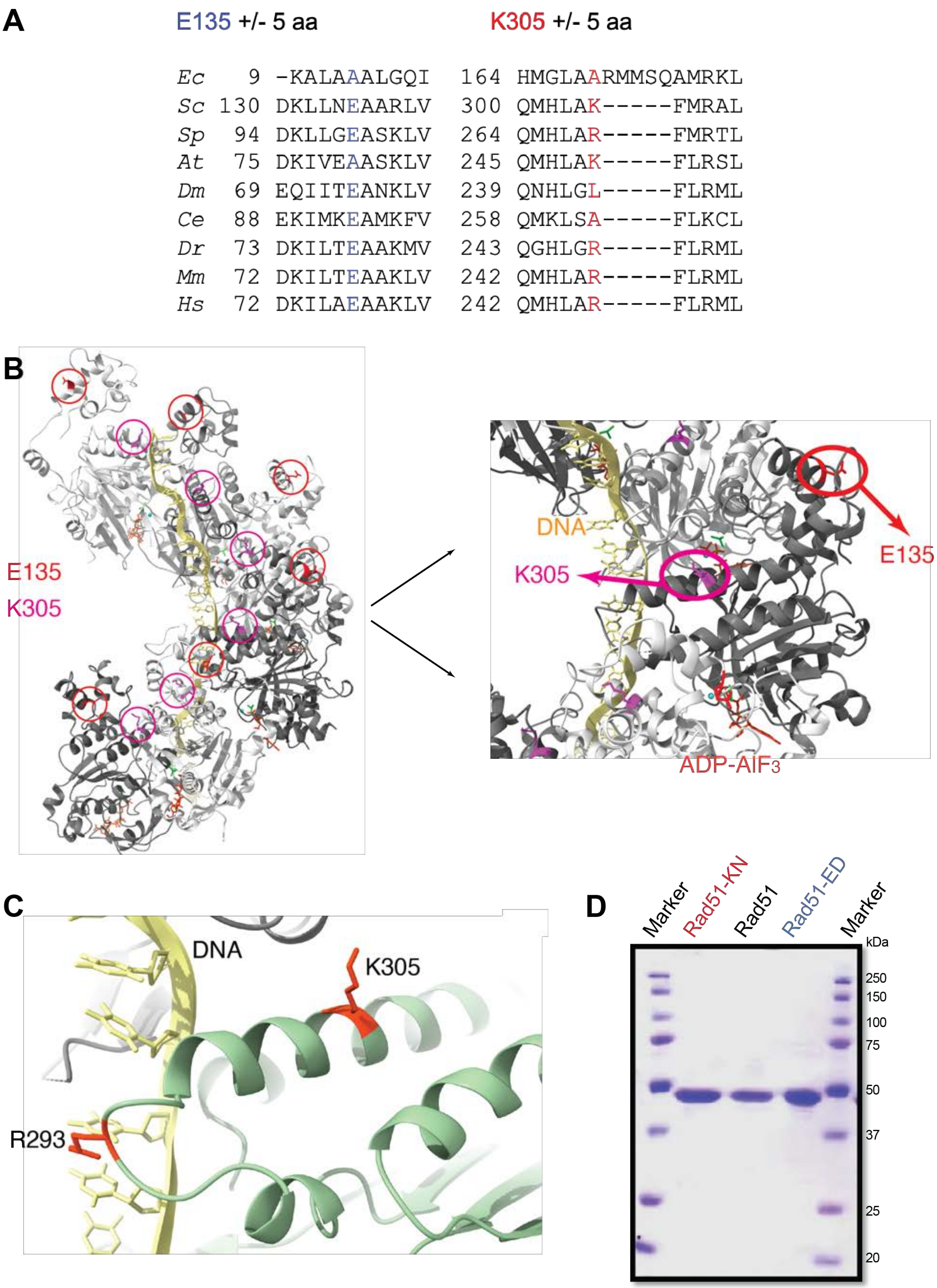
A
E135 +/- 5 aa
K305 +/- 5 aa
Ec 9 -KALAAALGQI 164 HMGLAARMMSQAMRKL
Sc 130 DKLLNEAARLV 300 QMHLAK-----FMRAL
Sp 94 DKLLGEASKLV 264 QMHLAR-----FMRTL
At 75 DKIVEAASKLV 245 QMHLAK-----FLRSL
Dm 69 EQIITEANKLV 239 QNHLGL-----FLRML
Ce 88 EKIMKEAMKFV 258 QMKLSA-----FLKCL
Dr 73 DKILTEAAKMV 243 QGHLGR-----FLRML
Mm 72 DKILTEAAKLV 242 QMHLAR-----FLRML
Hs 72 DKILAEAAKLV 242 QMHLAR-----FLRML
B
E135
K305
DNA
K305
E135
ADP-AlF3
C
DNA
K305
R293
D
Marker
Rad51-KN
Rad51
Rad51-ED
Marker
kDa
250
150
100
75
50
37
25
20

Supplementary Figure 5: Localization of E135 and K305 in the Rad51-ssDNA-ADP-AlF_3 structure and purification of Rad51-ED and Rad51-KN mutant proteins. A. Sequence conservation of Rad51-E135 and RAD51-K305 in comparison with *Escherichia coli* RecA (*Ec*) and Rad51 from *Saccharomyces cerevisiae* (*Sc*), *Schizosaccharomyces pombe* (*Sp*), *Arabidopsis Thaliana* (*At*), *Drosophila melanogaster* (*Dm*), *Caenorhabditis elegans* (*Ce*), *Dario rerio* (*Dr*), *Mus musculus* (*Mm*), and *Homo sapiens* (*Hs*). **B.** Overview of the Rad51-ssDNA-ADP-AlF3 structure (17) and positions of the E135 (red circle) and K305 (magenta circle). An enlarged view of E135 and K305 was shown on the right, relative to ssDNA (yellow), ADP (orange), AlF_3 (green), and Mg^{2+} (cyan). **C.** Detailed view of K305 in the alpha helix ending in R293, which intercalates with bases to enforce the triplet spacing of Rad51-bound DNA. **D.** *Saccharomyces cerevisiae* wild type Rad51, Rad51-KN, and Rad51-ED proteins were purified and 1 µg each was analyzed by 10 % SDS-PAGE and stained with Coomassie brilliant blue. Molecular markers are in the outer lanes. Source data are provided as a Source Data file.

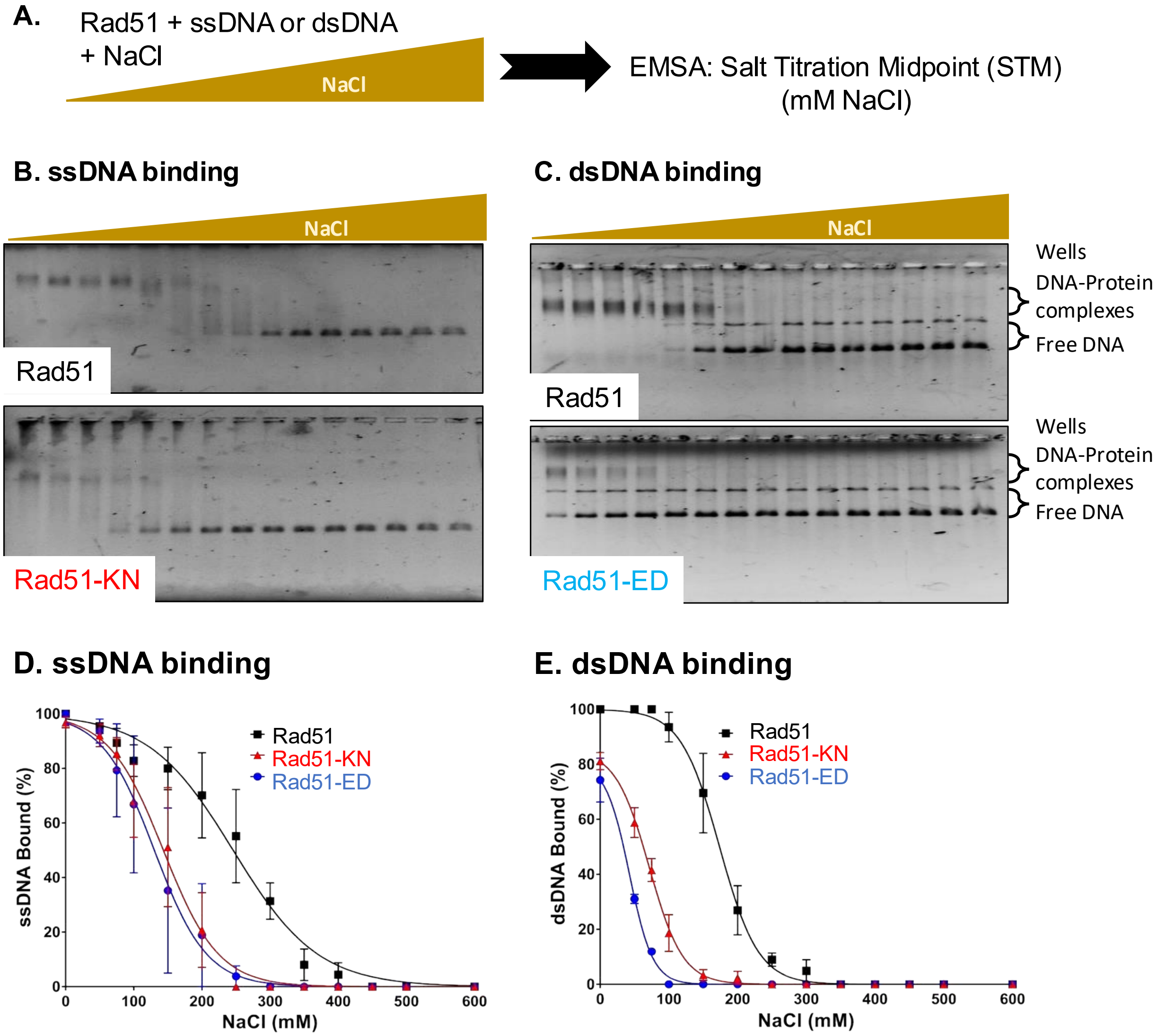

F. STM [mM NaCl]

	ssDNA	dsDNA
Rad51	246 ± 9	174 ± 4
Rad51-KN	143 ± 7	71 ± 3
Rad51-ED	130 ± 10	42 ± 3

Supplementary Figure 6. DNA binding defects of Rad51-ED and Rad51-KN in salt-midpoint titration assays. A. Reaction scheme of EMSA assays. **B, C.** Representative gels with ssDNA (B) and dsDNA (C). **D-F.** Salt titration midpoint (STM) DNA binding analysis by EMSA. The nucleoprotein complexes were assembled by incubating 3.33 µM of Rad51 proteins (Rad51, Rad51-ED, Rad51-KN) together with 10 µM nt of ssDNA (ΦX174 virion) or 10 µM of dsDNA ((ΦX174 RF1) in the presence of the indicated NaCl concentration for 15 min at 30 °C. Shown are means from n=4 (Rad51) and n=3 (Rad51-KN, Rad51ED), the error bars represent the standard error. The NaCl concentrations of the calculated STM are shown in **F**. Source data are provided as a Source Data file.

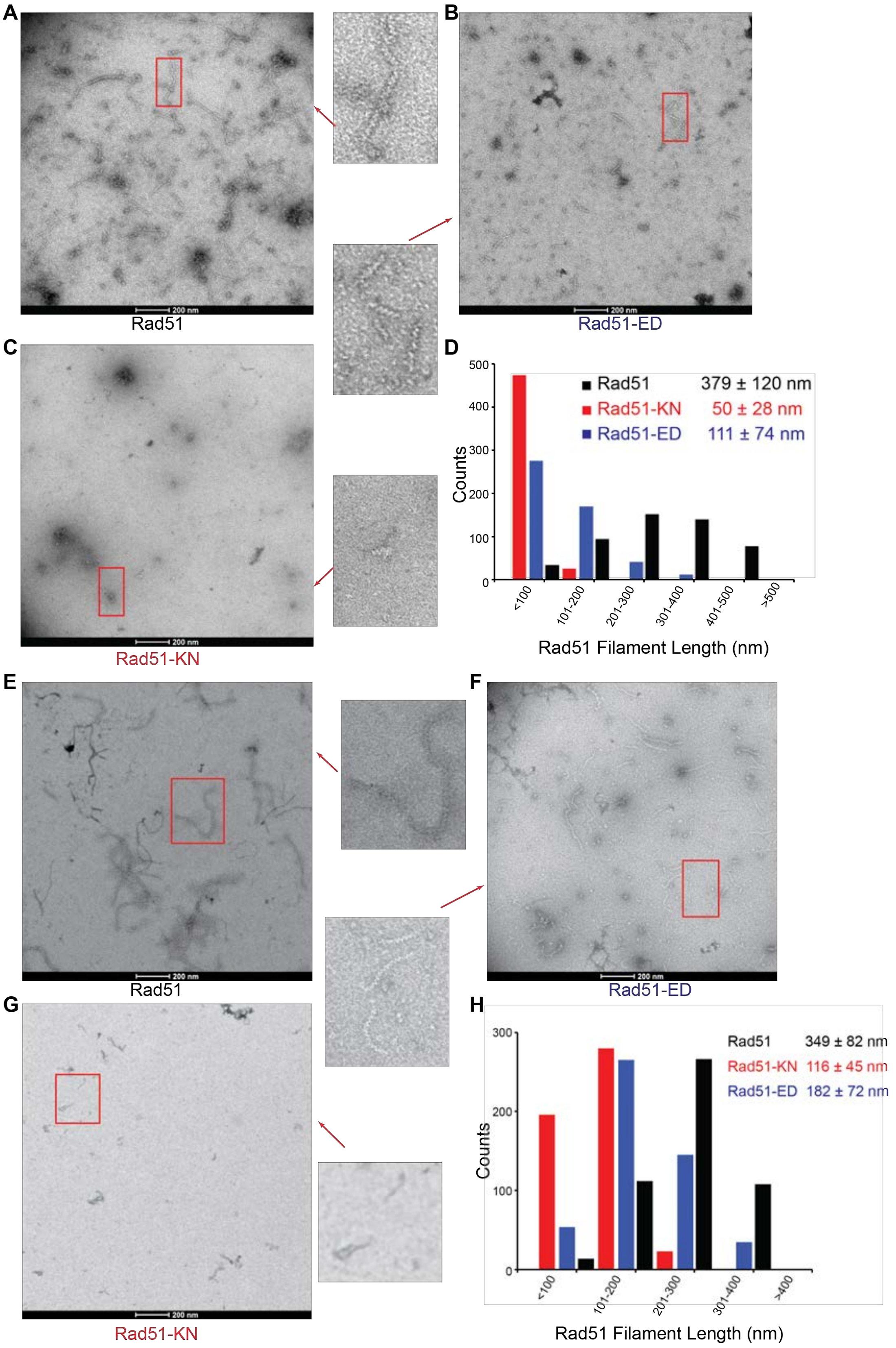
A
B
200 nm
Rad51
Rad51-ED
C
D
Rad51
379 ± 120 nm
Rad51-KN
50 ± 28 nm
Rad51-ED
111 ± 74 nm
Counts
500
400
300
200
100
0
<100
101-200
201-300
301-400
401-500
>500
Rad51 Filament Length (nm)
Rad51-KN
E
F
Rad51
Rad51-ED
G
H
Rad51
349 ± 82 nm
Rad51-KN
116 ± 45 nm
Rad51-ED
182 ± 72 nm
Counts
300
200
100
0
<100
101-200
201-300
301-400
>400
Rad51 Filament Length (nm)
Rad51-KN

Supplementary Figure 7. Rad51-ED and Rad51-KN form much shorter filaments on dsDNA than on ssDNA compared to Rad51. A-C, Representative images of negatively stained filaments of Rad51 (**A**), Rad51-ED (**B**), and Rad51-KN (**C**) on dsDNA. (**D**) Quantification of filament length analysis. For each reaction condition, 500 filaments were measured (2.5 μM Rad51, Rad51-ED, or Rad51-KN, and 7.5 μM 1-kilobase dsDNA). **E-G**, Representative images of negatively stained filaments of Rad51 (**E**), Rad51-ED (**F**), and Rad51-KN (**G**) on ssDNA. (**H**) Quantification of filament length analysis. For each reaction condition, 500 filaments were measured (2.5 μM Rad51, Rad51-ED, or Rad51-KN, and 7.5 μM 700 nt ssDNA). The means ± 1 standard deviation, and distribution of filament lengths are shown. Scale bars, 200 nm. The red box indicates an enlarged region of filament species with different lengths. Source data are provided as a Source Data file.

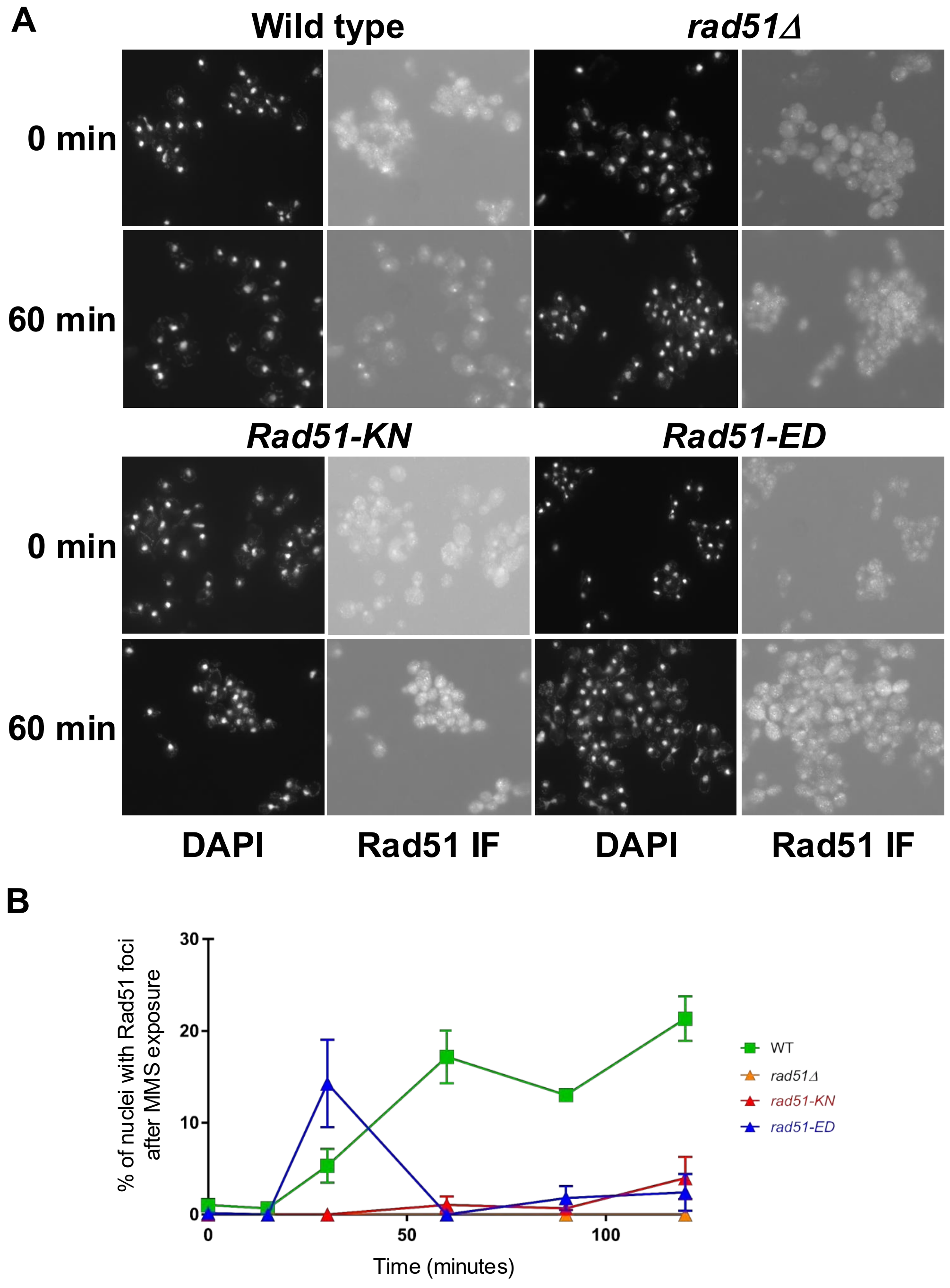

Supplementary Figure 8. Rad51 focus formation defect in response to MMS treatment in *rad51-ED* and *rad51-KN*. Wild type (WDHY1636), *rad51Δ* (WDHY2542), *rad51-ED* (WDHY3548), and *rad51-KN* (WDHY3962) were treated with 0.1% MMS for 1 hr prior to detecting Rad51 foci by immunofluorescence at the indicated times. Shown are means for n=4-6 ± 1 SEM ,165-542 cells were evaluated per time point. Source data are provided as a Source Data file.

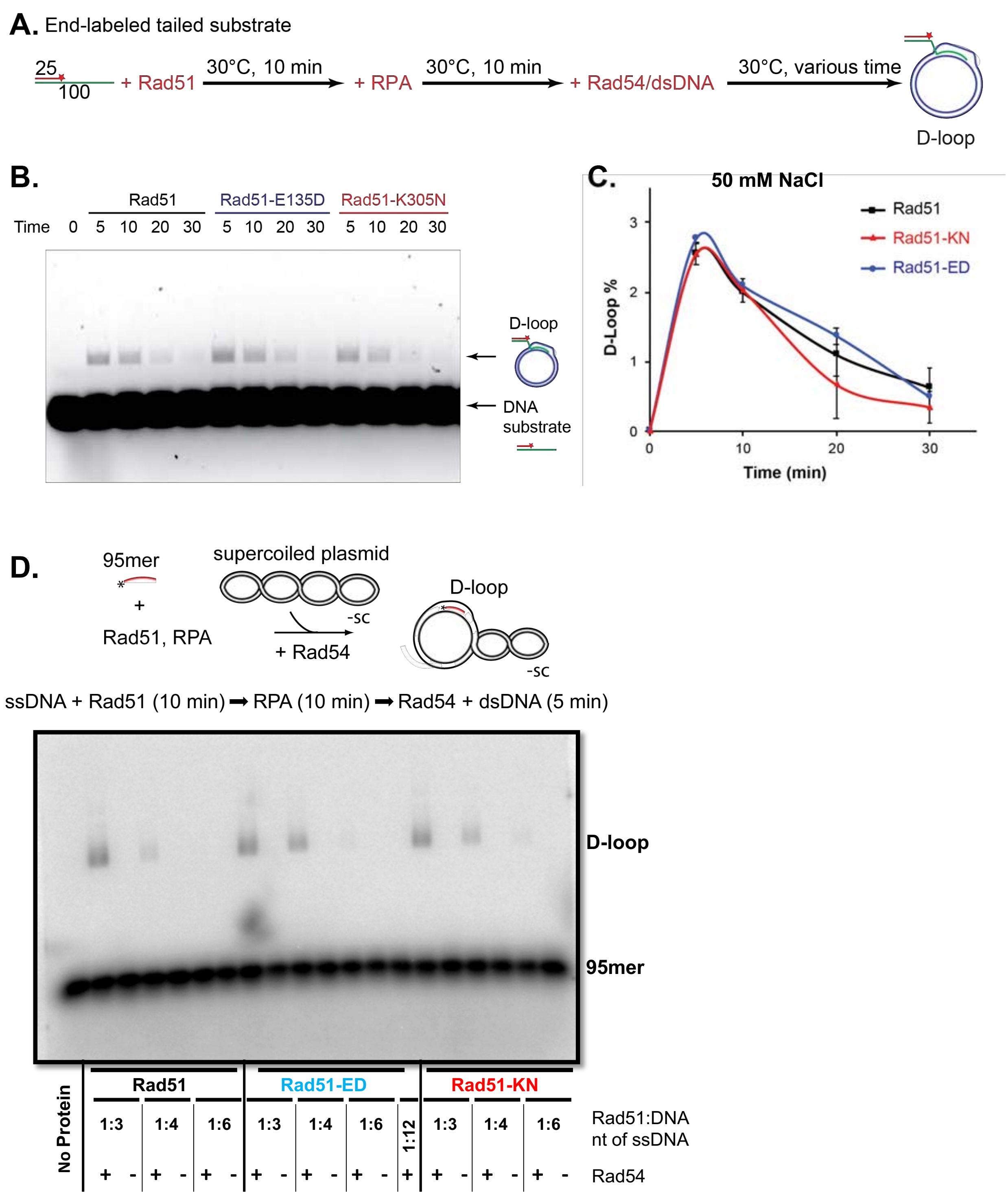

Supplementary Figure 9. D-loop formation by Rad51-ED and Rad51-KN. A. Schematic representation of the D-loop assay design. A fluorescently labeled tailed substrate was incubated with Rad51, Rad51-ED, and Rad51-KN, then combined with RPA and Rad54+dsDNA, and incubated as illustrated. **B**. Gel image showing D-loop formation by Rad51 species in a low-salt buffer. **C**. Quantification of D-loop yield in **B**. Plotted are the means ± 1 sd

from n = 3. **D.** Qualitative D-loop assays with 5'-end labelled 95mer (oIWDH566, see Table S3) and pUC19 dsDNA conducted for 5 min. Products were analyzed by gel electrophoresis and a representative gel is shown. The reactions were conducted at the Rad51 to nucleotide (nt) ssDNA ratios indicated in the presence and absence of Rad54. Source data are provided as a Source Data file.

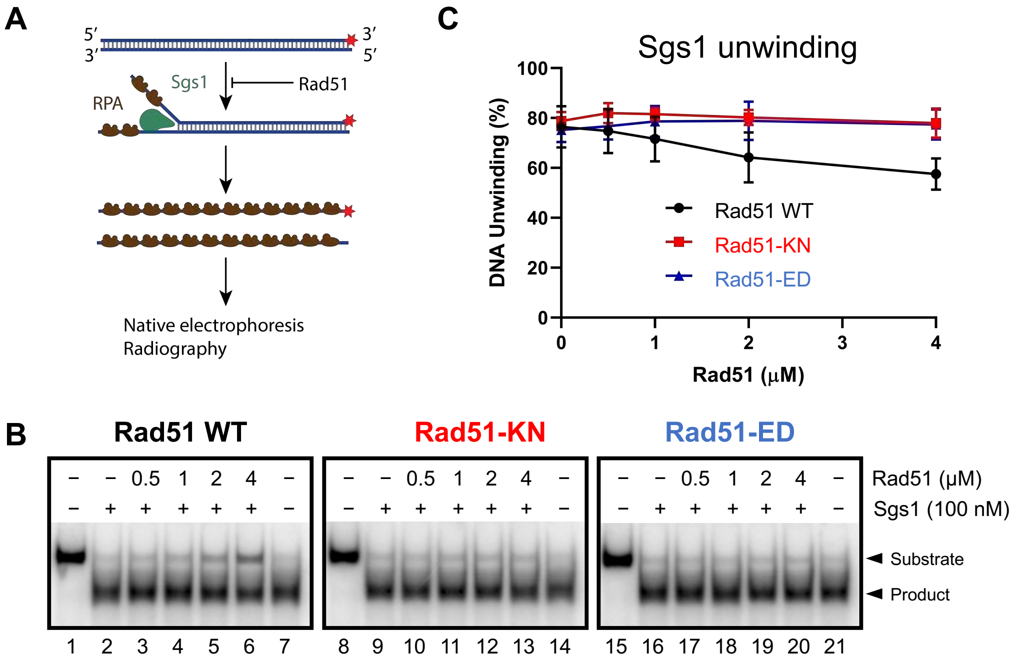

Supplementary Figure 10: Rad51-KN and Rad51-ED are defective in protecting dsDNA from unwinding by Sgs1. A. Cartoon depicting the helicase assay with Sgs1 performed in panels B-C. The red asterisks represent the position of the radioactive label. **B.** Representative helicase assays with Sgs1 in the presence of increasing concentrations of Rad51 wild type and variants. In lanes 7, 14 and 21, the substrate was boiled to indicate the position of the ssDNA. **C.** Quantitation of experiments such as shown in B. Averages shown; error bars, range; n=2. Source data are provided as a Source Data file.

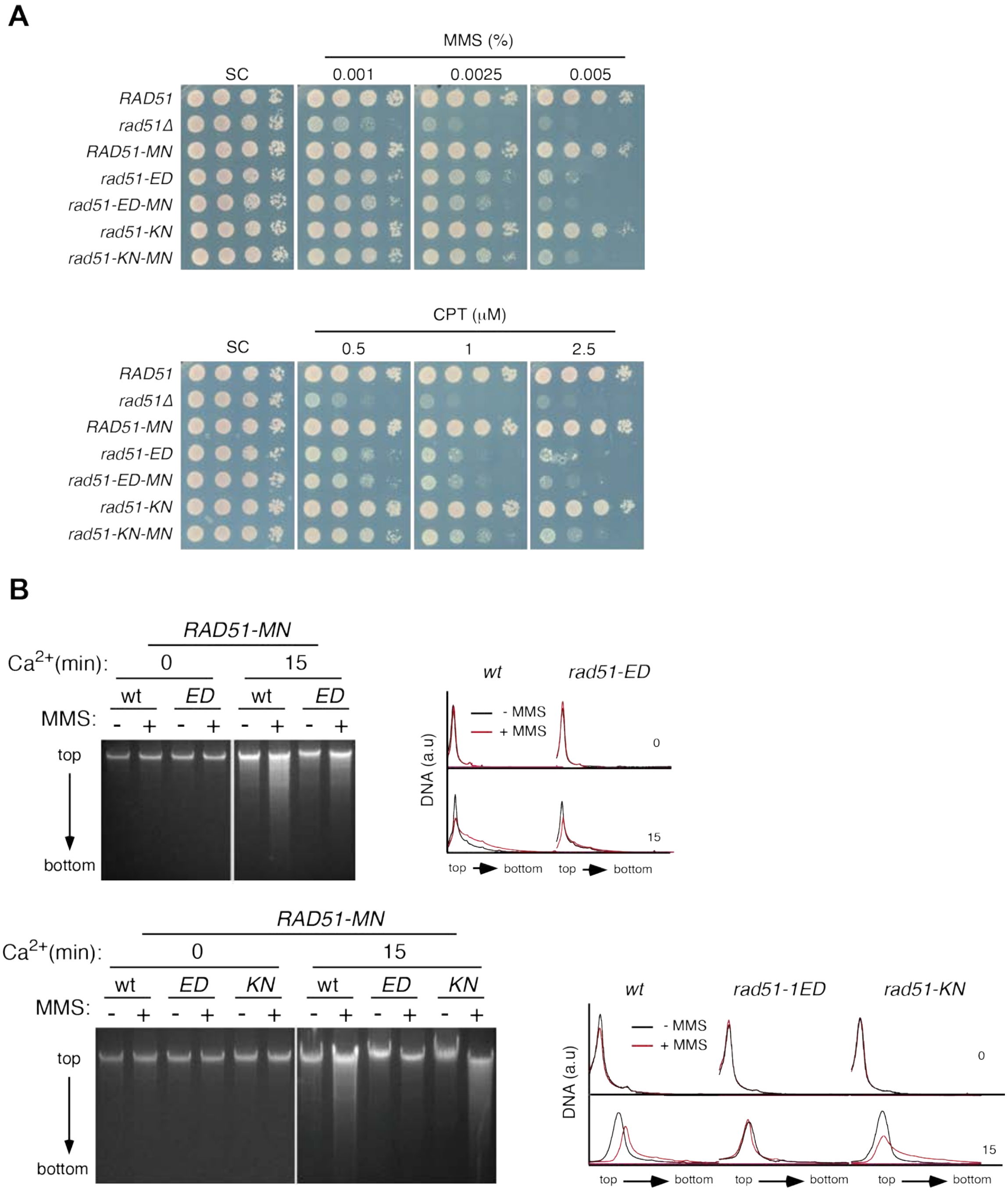

Supplementary Figure 11. Control and repeat experiments for Figure 10. A. MN tag does not alter the sensitivity profile for *rad51-ED* but does so for *rad51-KN*. Serial dilutions of *RAD51-MN* (wt) (wR51MN-2), *rad51-ED-MN* (wR51-135MN) *and rad51-KN-MN* (wR51-305MN) cells grown on plates containing the indicated DNA damaging agent and concentrations. **B.** Two repeat experiments for Figure 10B. Source data are provided as a Source Data file.

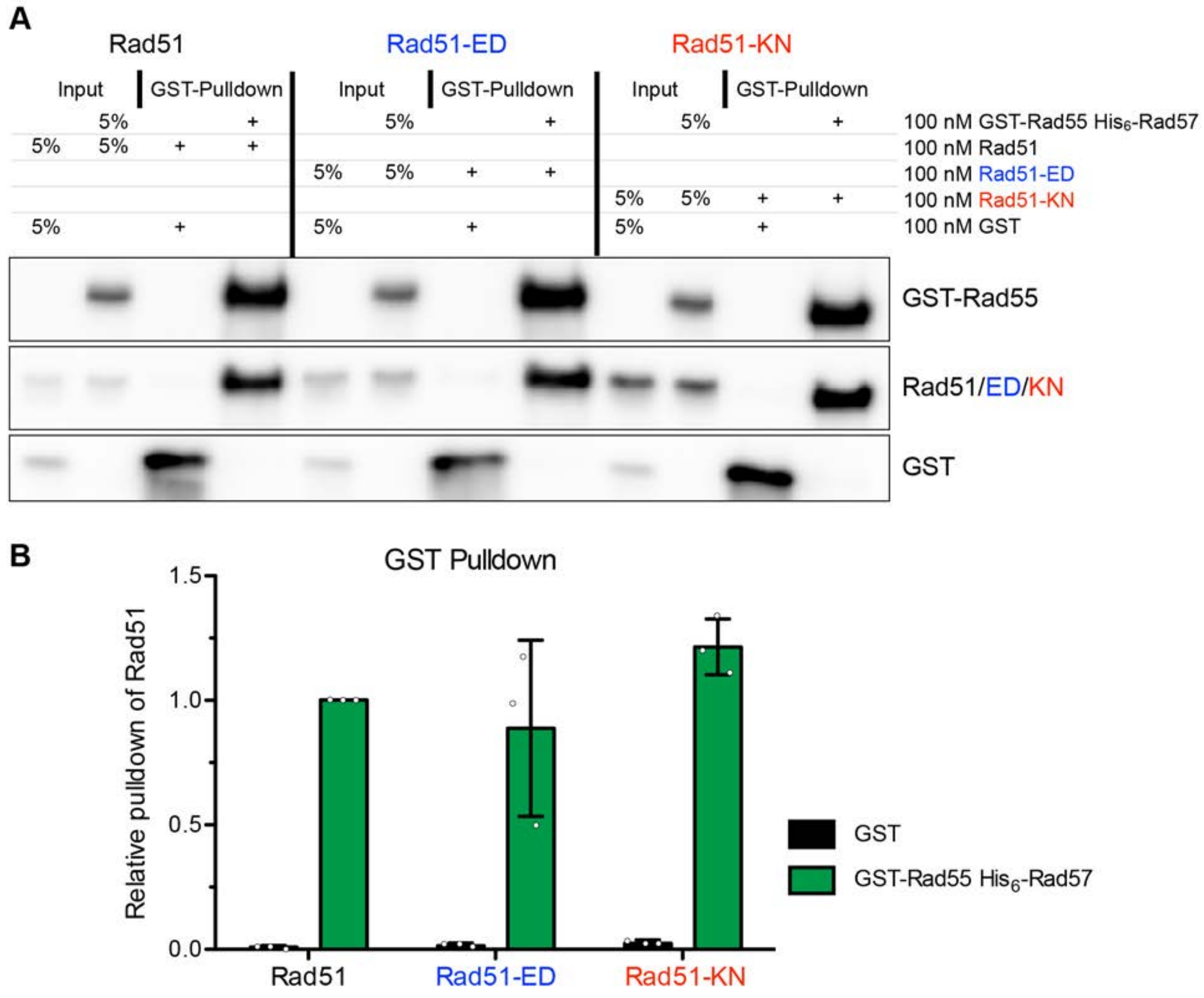

Supplementary Figure 12. Rad51-ED and Rad51-KN are proficient in their interaction with Rad55-Rad57. A. Representative immunoblot of pulldown of Rad51 by GST-tagged Rad55 in complex with Rad57. **B.** Quantitation of pulldowns from n=3, shown are means with error bars representing 1 standard deviation. Source data are provided as a Source Data file.

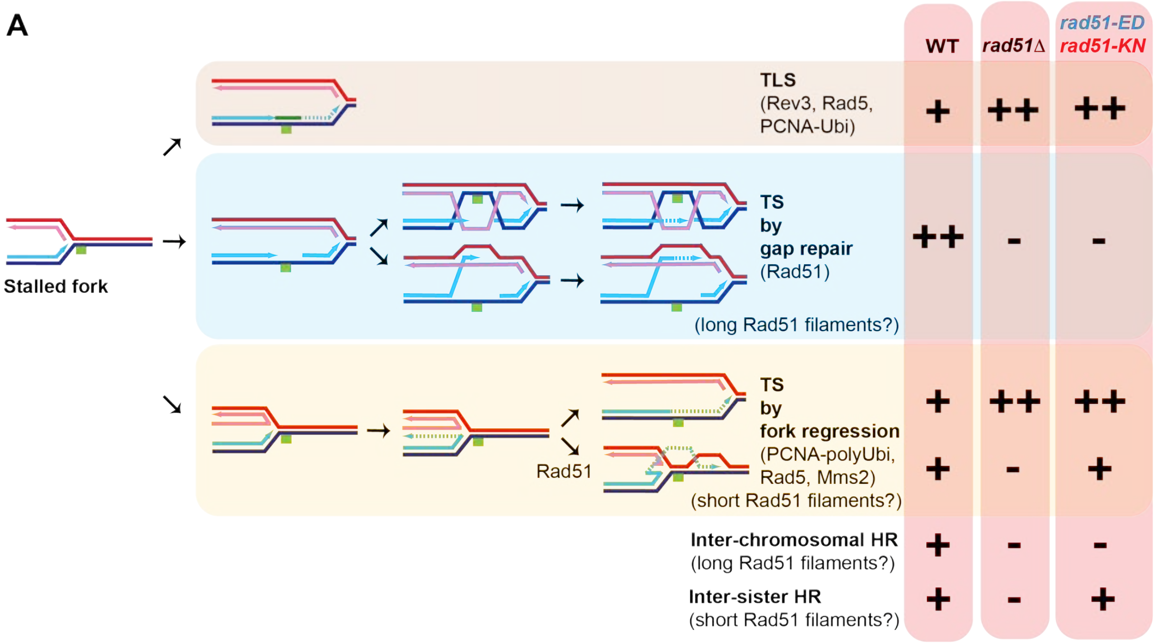

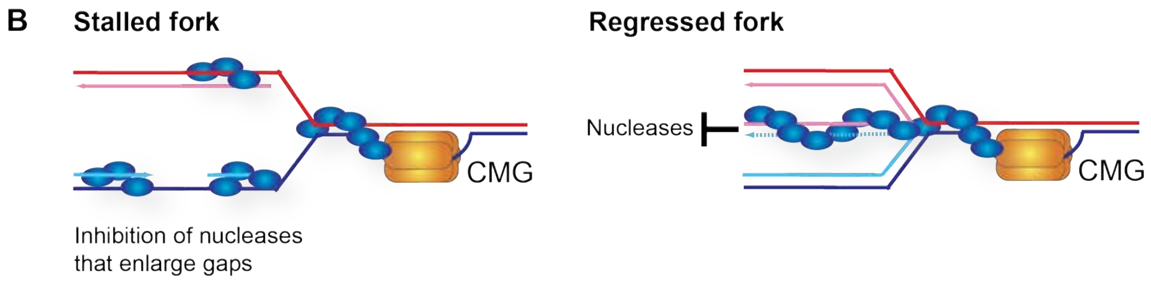

Supplementary Figure 13. Summary, interpretation, and model. A. Representation of postreplication repair pathways and genes analyzed in this study, including translesion synthesis (TLS) which in budding yeast depends on PCNA ubiquitylation, Rad5, and Rev3. There are two different template switching (TS) pathways. First, an homologous recombination (HR)-dependent pathway of gap repair that can proceed either by gap invasion (top) or end invasion (bottom). Both pathways are expected to be dependent on Rad51. Second, fork regression which in budding yeast is expected to depend on PCNA poly-ubiquitylation, Rad5, and Mms2 in analogy to mammalian cells. After processing of regressed forks by exonucleases, the resulting 3'-OH ending single-strand may form a filament and invade duplex DNA on the other side of the stalled fork. Based on the phenotypes of the *rad51-ED* and *rad51*-KN, we speculate that this type of template switch involves short nucleoprotein filaments, whereas gap repair between sisters and inter-chromosomal HR requires longer Rad51 nucleoprotein filaments. **B.** Potential sites of dsDNA binding by Rad51 to protect from exonucleolytic degradation of gaps at stalled forks and the dsDNA end of the regressed fork. For more discussion see text.
