# Supplementary material for: Rad51 determines pathway usage in post-replication repair
Source: Nat Commun. 2026 Jan 10;17:1359. doi: 10.1038/s41467-025-68109-1 (PMC12876063; doi:10.1038/s41467-025-68109-1)

Figure 3H

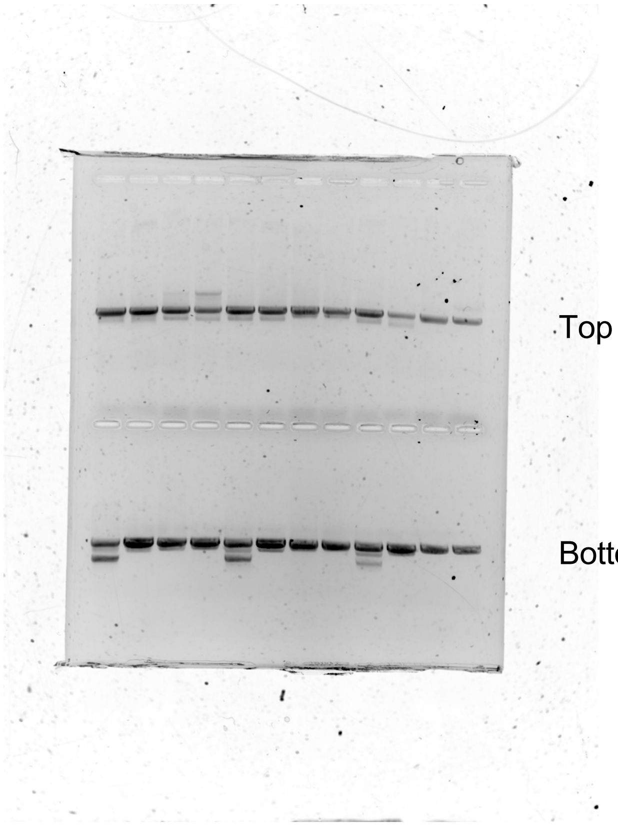

Top gel

Bottom gel

Figure 4A

Left Right

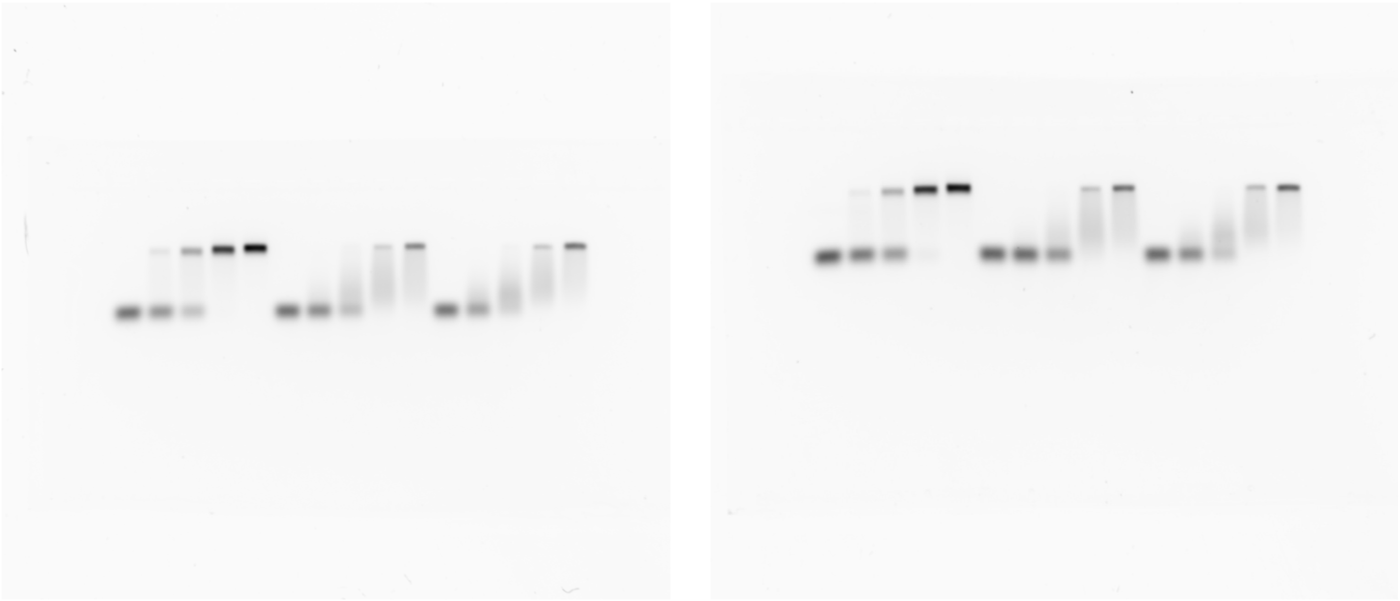

Figure 4B

Left Right

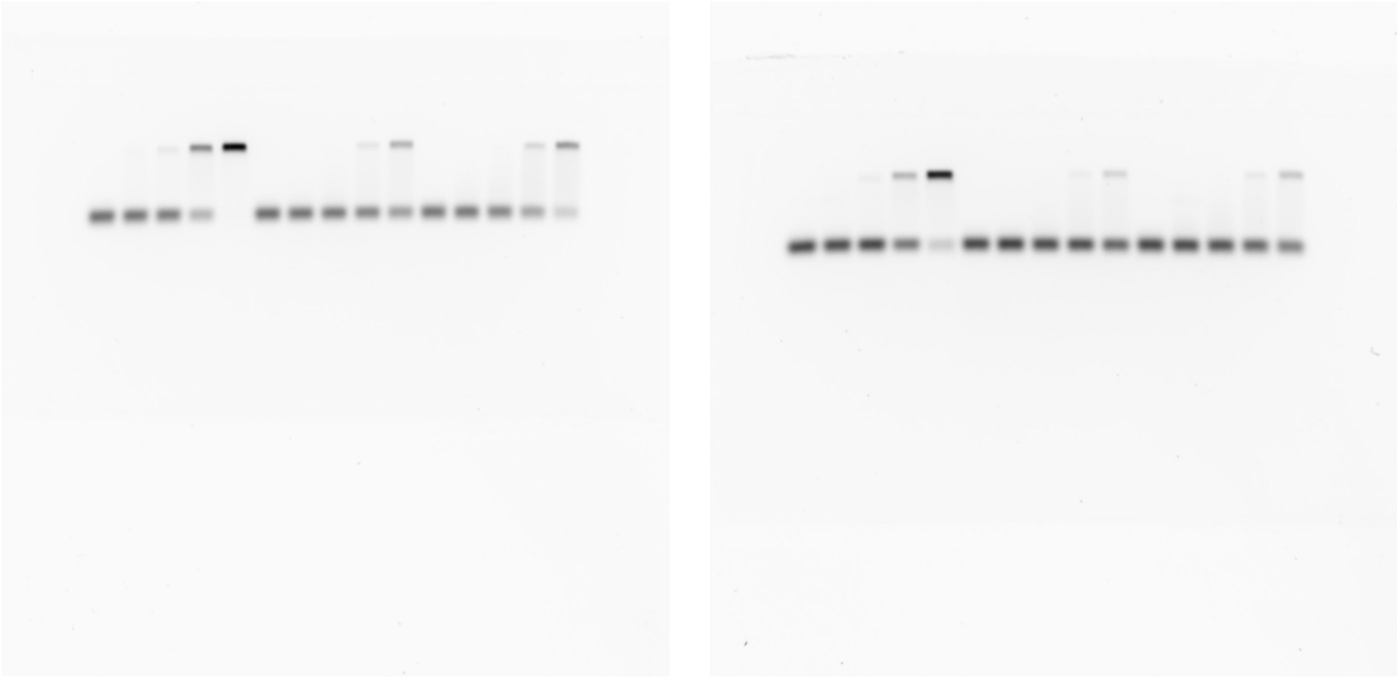

Figure 5D

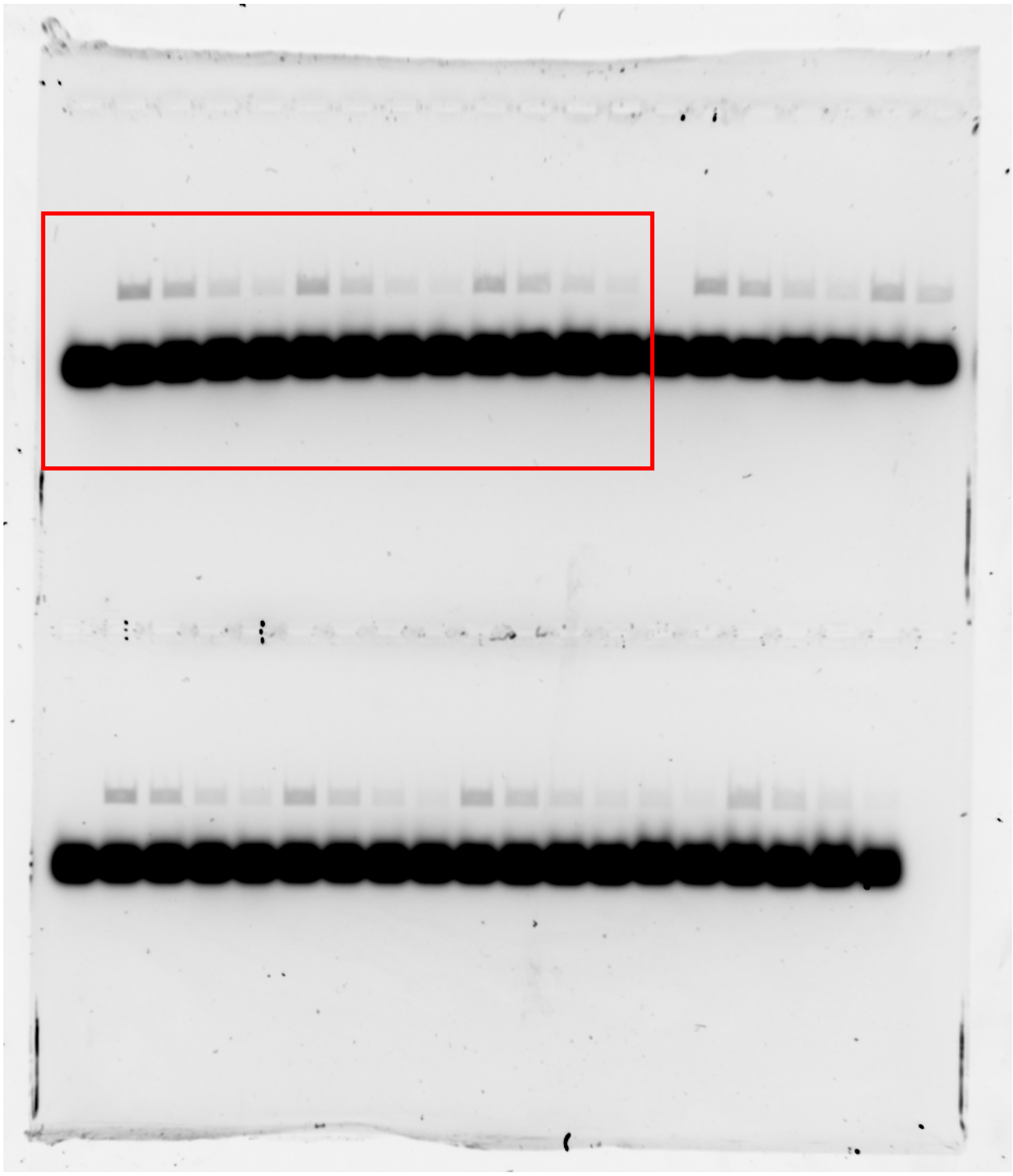

Figure 6B

Left Middle Right

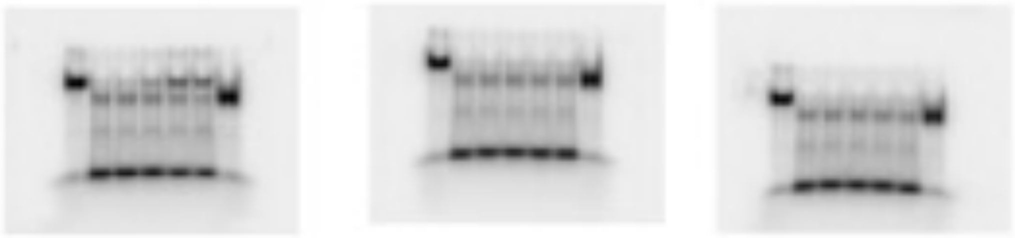

Figure 6E

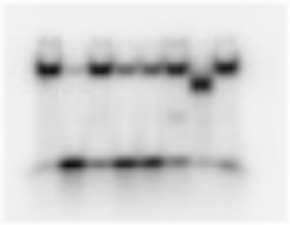

Figure 10A

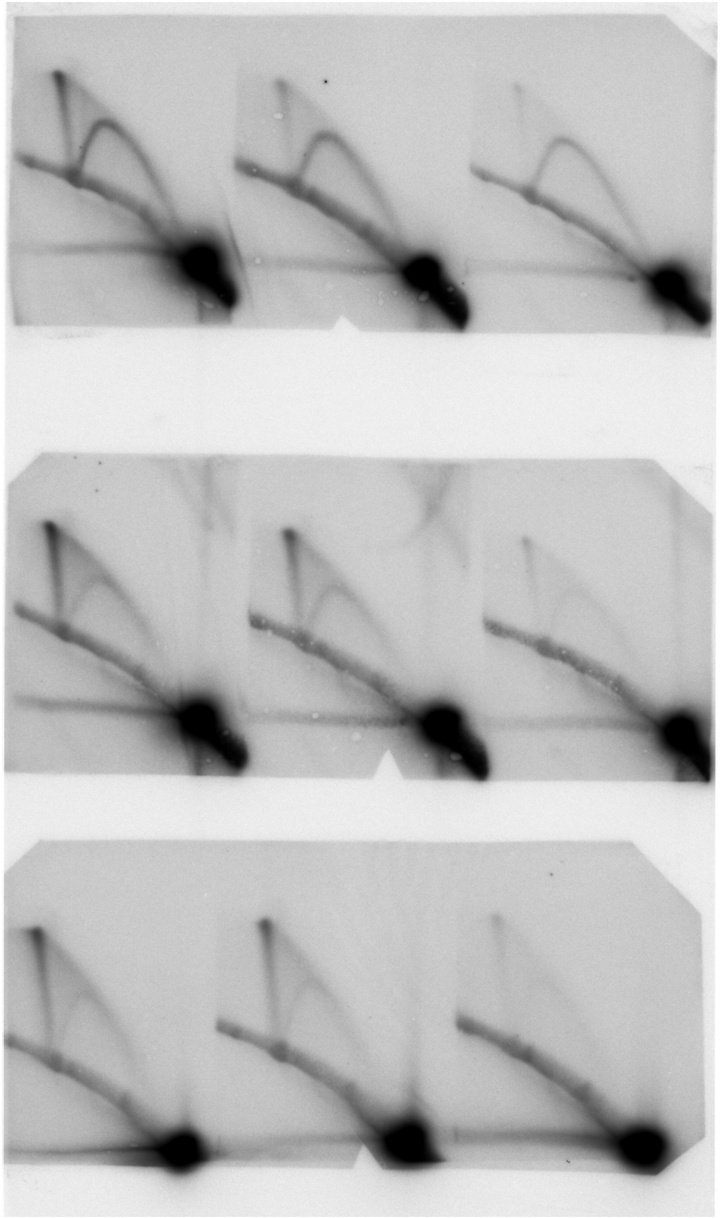

Figure 10B

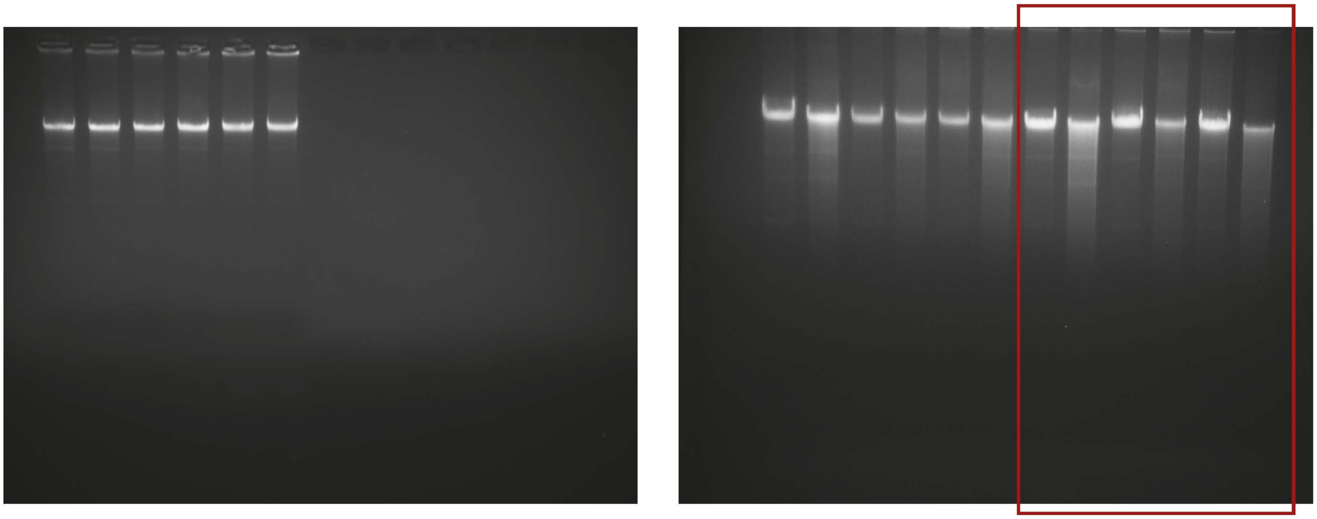

Supplementary Figure 1

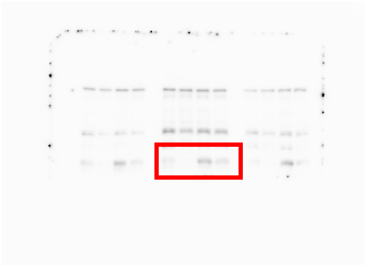

Rad51

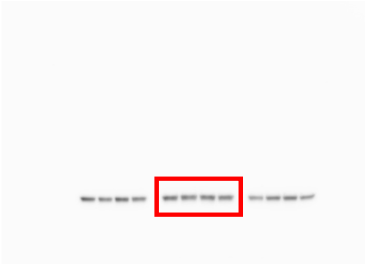

GAPDH

Supplementary Figure 5D

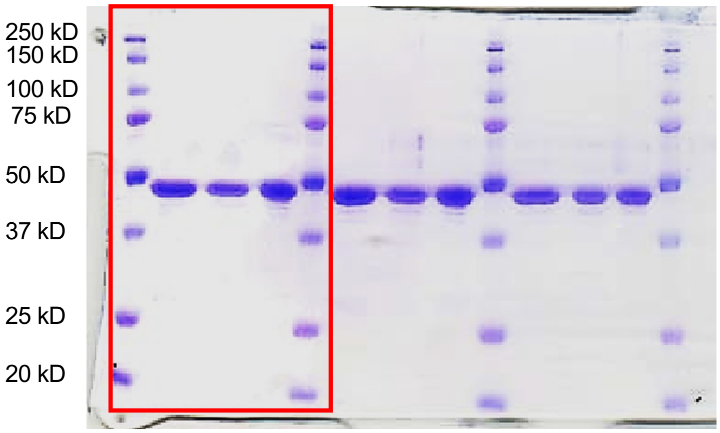

Supplementary Figure 6B

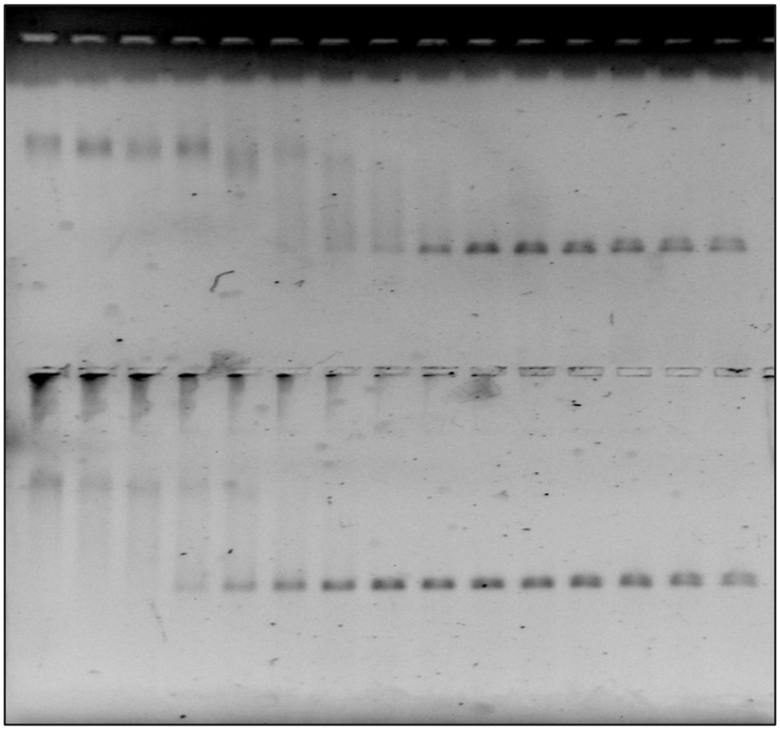

Supplementary Figure 6C

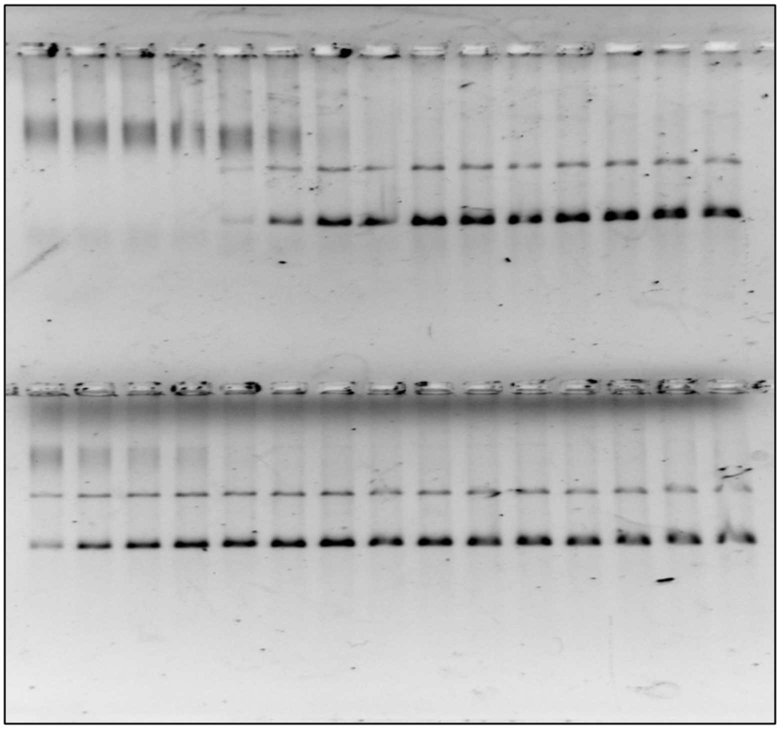

Supplementary Figure 9B

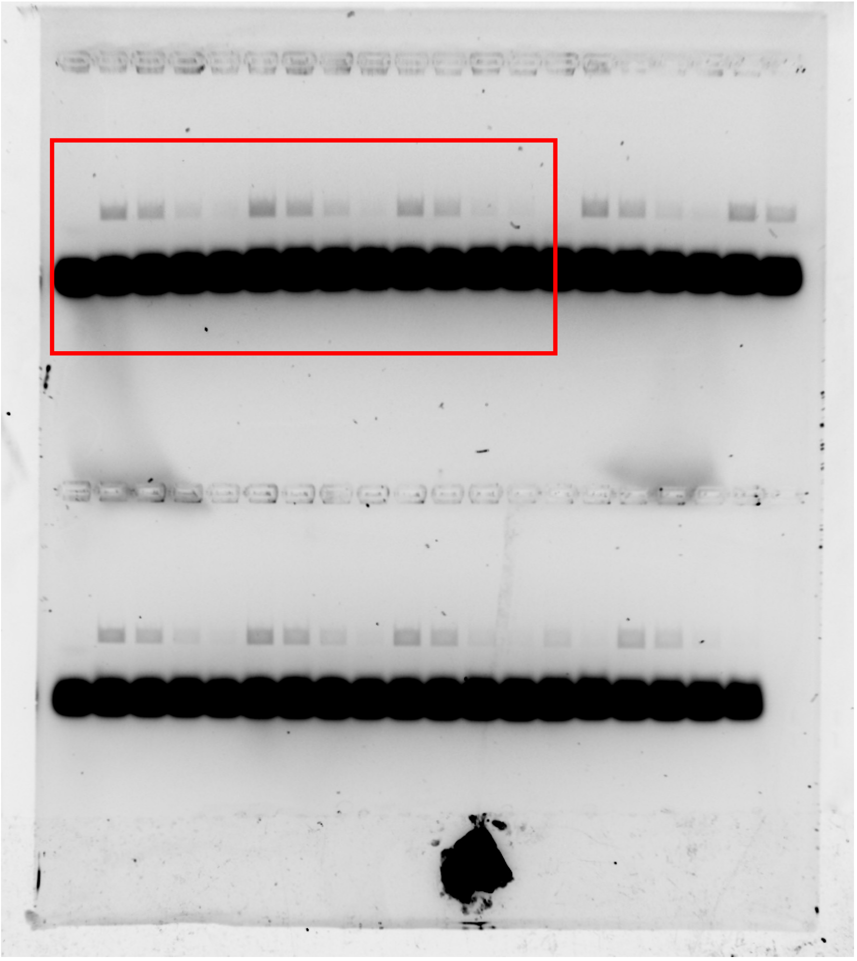

Supplementary Figure 9D

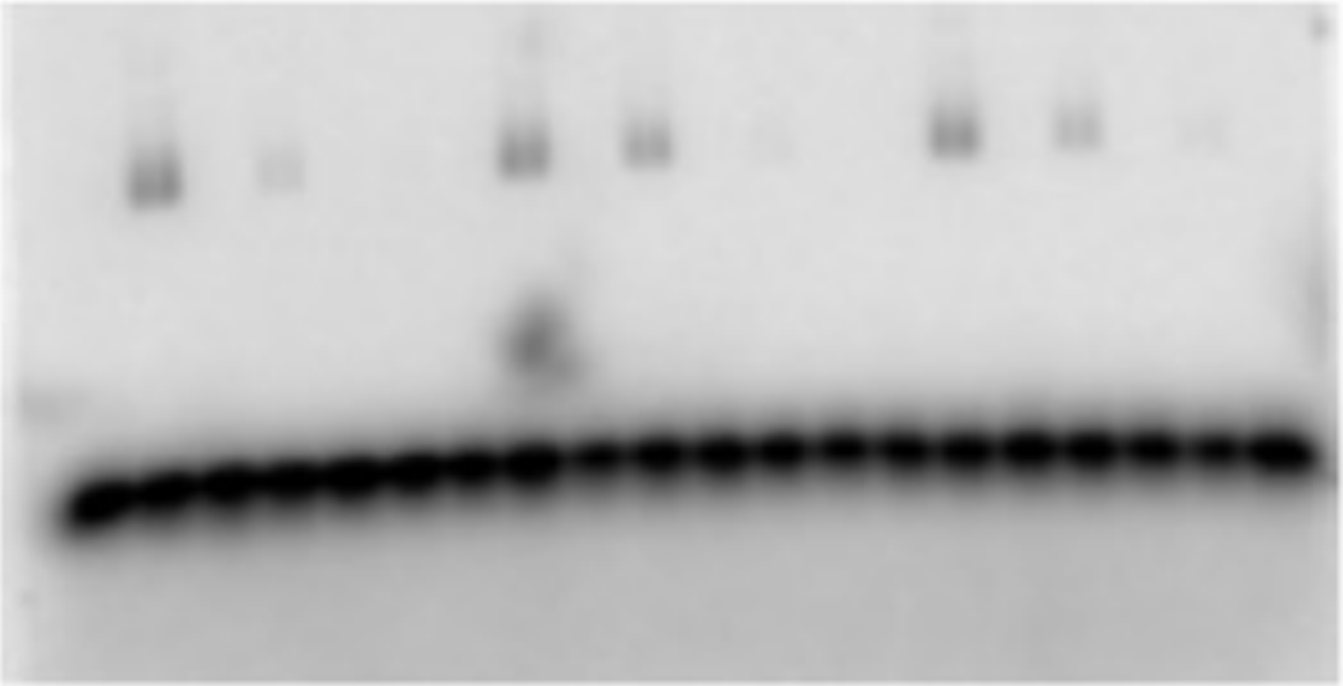

Supplementary Figure 10B

Left

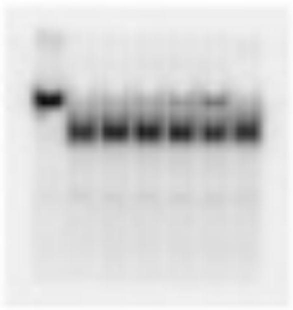

Middle

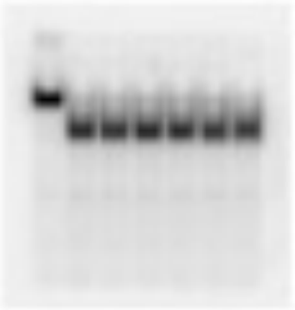

Right

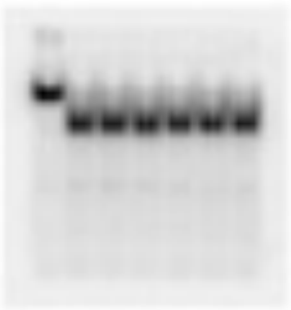

Supplementary Figure 11B

Top left

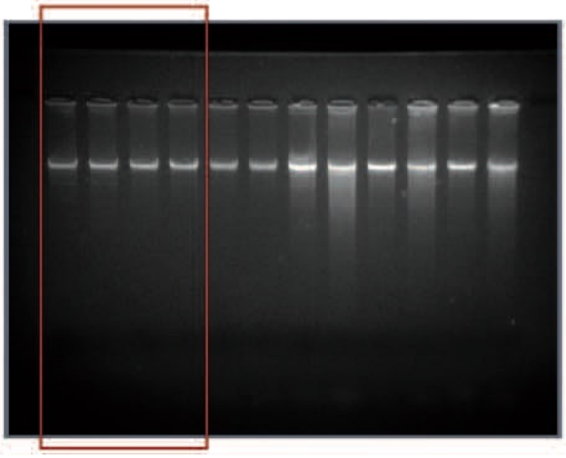

Top Right

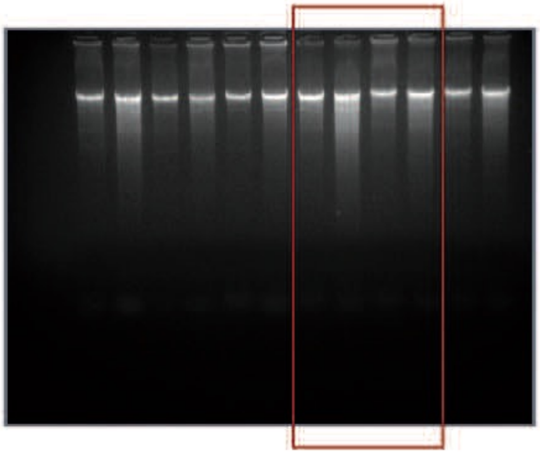

Bottom left

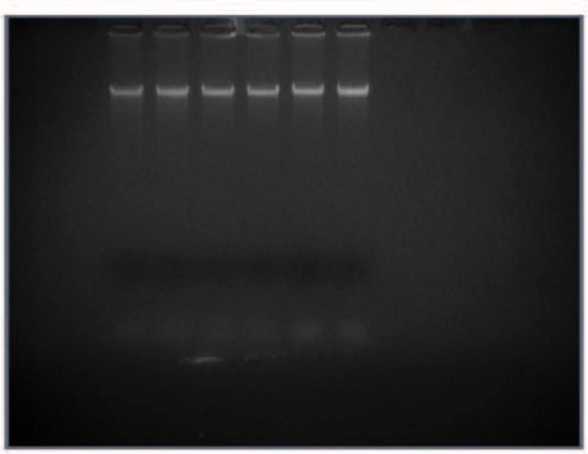

Bottom Right

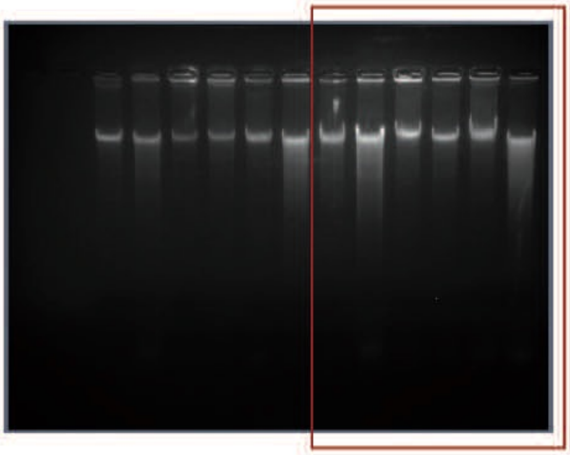

Supplementary Figure 12A

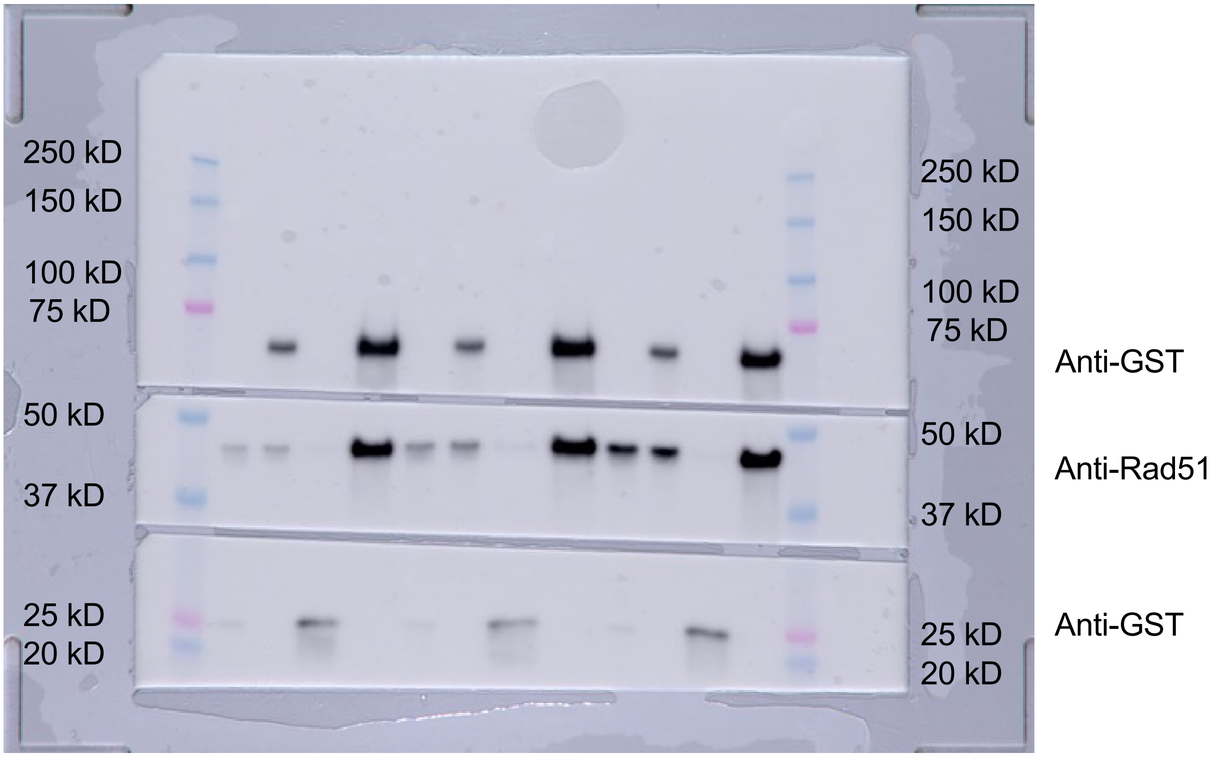

Supplement: Supplementary file 3 — Source Data [file 41467_2025_68109_MOESM3_ESM.zip › 41467_2025_68109_MOESM3_ESM/Source_data_gel_images.pdf]
